# The Chemistry From Tin Iodide Molecular Inks to FASnI_3_ Nanocrystals

**DOI:** 10.1002/smll.202511842

**Published:** 2026-02-06

**Authors:** Kushagra Gahlot, Julia N. Kraft, Manuel Pérez‐Escribano, Mihai T. Todosia, Karla Ravin, Joaquín Calbo, Loredana Protesescu

**Affiliations:** ^1^ Zernike Institute for Advanced Materials University of Groningen Groningen The Netherlands; ^2^ Instituto de Ciencia Molecular Universitat De València Paterna Spain

**Keywords:** ^119^Sn NMR spectroscopy, Formamidinium Tin Iodide, Lead‐free Perovskites, Perovskite Nanocrystals

## Abstract

The controlled synthesis of high‐performance tin halide perovskite nanostructures hinges on the coordination chemistry that governs precursor speciation within molecular inks. Here, we elucidate the complexation dynamics of SnI_2_ with two benchmark Lewis bases widely used in nanocrystal syntheses: a strong primary amine (R–NH_2_) and a weaker substituted phosphine (R’_3_–P). Correlated in situ ^1^ ^1^ ^9^Sn NMR and UV–Vis absorption spectroscopy, supported by density functional theory calculations, reveal that both ligands (L) form monomeric SnI_2_–L adducts, with R–NH_2_ consistently exhibiting stronger coordination than R_3_′–P, as quantified by the intrinsic bond strength index and interaction energies. Higher ligand loadings destabilize SnI_2_–L_x_ complexes, particularly for phosphines, whereas we show that amine‐bound multimeric (SnI_2_)_x_(R–NH_2_)_x_ (x = 2−3) species can be present at low ligand concentrations. These molecular‐level insights directly correlate with nanocrystal formation pathways. Stronger Sn─N coordination drives the emergence of 2D Ruddlesden–Popper phases, while weaker Sn–P interactions favor bulk‐like 3D FASnI_3_ nanocrystals due to insufficient stabilization of early‐stage intermediates. Guided by this understanding, an amine‐free, three‐precursor strategy employing a strong zwitterionic ligand enables phase‐pure 3D FASnI_3_ nanocrystals with improved optical and colloidal stability. This work establishes a predictive framework for designing robust molecular inks for tin halide perovskites and perovskitoid nanostructures.

## Graphical abstract

The complexation dynamics of SnI_2_ with two benchmark Lewis bases (oleylamine and trioctylphosphine) at different ligand loadings, as well as an amine‐free zwitterionic approach, were directly correlated with the formation of different perovskite nanostructures, establishing a predictive framework for designing robust molecular inks for tin halide perovskite and perovskitoid nanostructures.

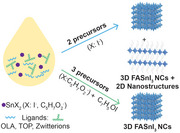

## 1 Introduction

Molecular inks play a key role in the formation of metal halide perovskites, whether in single‐crystal, nanocrystal (NC), or thin film formation [1, 2, 3, 4, 5]. These inks, composed of reactive metal complexes, are formed via the coordination of the metal halide precursors (e.g. PbI_2_, SnI_2_) with a coordinating Lewis base ligand [6]. The nature and strength of the interactions between the metal centre and coordinating species have shown direct influence on the crystallization pathway [6], dimensionality [7], and properties of the final material [8], thus, a key understanding of their interactions is essential for rational ink design. Tin halide salts, especially SnI_2_, are the primary tin precursor in the synthesis of different tin perovskite nanostructures, serving as both the tin and halide source [9, 10]. The empty p‐orbital on Sn imparts Lewis acid character to these metal salts, hence, they easily form adducts with Lewis bases, making them key candidates for the development of molecular inks.

In thin film synthesis, polar solvents such as dimethylformamide (DMF), dimethyl sulfoxide (DMSO), dimethylpropyleneurea (DMPU) and hexamethylphosphoramide (HMPA), are commonly employed as the Lewis base as their donacity (expressed using the donor number (DN)) can be varied to tune the strength of the interactions with Sn. This directly influences the film formation pathway, with lower DN solvents forming weaker adducts with SnI_2_. For example, complexes and adducts of SnI_2_ formed via the interaction with solvents with a lower DN, such as DMF (DN: 29.8 kcal mol^−1^) have shown the formation of inhomogeneous films plagued by small grains, attributed to the formation of small nuclei, resulting in a high nucleation density at the crystallization point [6, 11, 12]. Conversely, employing stronger coordinating solvents, such as HMPA (38.8 kcal mol^−1^), has been shown to promote the formation of larger nuclei, thereby facilitating the growth of larger grains and uniform films [6, 13].

In non‐polar systems, commonly used for NCs synthesis, the reaction (nucleation and growth) is performed in non‐coordinating solvents (such as octadecene or squalene), facilitated by long‐chain organic ligands such as oleic acid (R–COOH), oleylamine (R–NH_2_), and trioctylphosphine (R_3_’‐P) serving as the Lewis bases to form adducts with the Sn source. Thus, tuning the nature of the ligand by varying the Lewis basicity (strong for primary amines with a pKa ∼ 10.7 [14], and weaker for substituted phosphines with pKa ∼9) [15] allows for control over the strength of tin iodide–ligand interactions. This directly impacting the nucleation and growth mechanism of the resulting NCs as well as their subsequent colloidal stability [16]. [7] Among the final products generated using tin halide inks, FASnI_3_ (FA = formamidinium) has emerged as a promising perovskite material, showing high optoelectronic potential in near infrared (e.g., 24% power conversion efficiencies in solar cells). [17] Synthetic methods to obtain colloidal FASnI_3_ NCs employ similar routes imported from their analogous lead‐based counterparts. These often use multiple Lewis bases such as R‐NH_2_ and R_3_’‐P concomitantly; however, understanding the effect of these ligands in the nucleation and growth mechanism of the resulting NCs and the components of the molecular inks still needs to be explored [18, 19, 20]. Notably, a recent study on precursor chemistry in the well‐explored CsSnI_3_ NC system shows that even subtle variations in precursor reactivity can significantly affect the resulting NC properties. This underscores the value of carefully selecting precursors with appropriate reactivity profiles [21]. At the same time, it also reveals how much remains to be understood regarding how ligand identity shapes precursor speciation and the dynamic coordination equilibria established prior to nucleation. Although some progress has been made, comprehensive studies on the reactivity of widely used capping ligands—such as oleylamine (R‐NH_2_) and trioctylphosphine (R_3_’‐P) and their direct influence on precursor evolution are still missing. Gaining these insights will be crucial for disentangling the competing pathways that dictate phase selectivity and dimensionality, ultimately enabling more precise control over nucleation, growth, and morphology when employing these workhorse ligands and developing novel synthetic methods for FASnI_3_ NCs.

Herein, we systematically investigated the complexation of SnI_2_ with benchmark Lewis base ligands R‐NH_2_ and R_3_’‐P. Using correlated in situ nuclear magnetic resonance (NMR) and UV–Vis absorption spectroscopy experiments, we were able to follow the evolution of the resulting SnI_2_ – Lewis base adducts over various molar ratios. We employed theoretical calculations to explain the phenomena further, and we confirm that both R‐NH_2_ and R_3_’‐P bind to SnI_2_ to form stable monomeric adducts, with R‐NH_2_ exhibiting consistently stronger Sn─N coordination than the Sn─P bonding in R_3_’‐P. Moreover, we reveal that higher ligand loading (n, m ≥ 2, where n and m are molarity of the R‐NH_2_ and R_3_’‐P, respectively) destabilizes the complexes—especially for R_3_’‐P. On the other hand, only R‐NH_2_‐based multimeric (SnI_2_)_x_(R‐NH_2_)_x_ (x = 2−3) species are predicted relatively stable, which are expected to be present at low ligand concentration. These inks were used as pre‐injection mixtures in the room temperature synthesis of FASnI_3_ NCs, our model system, allowing us to determine the role of these adducts in the formation of these structures. We found that when the stronger Lewis base R‐NH_2_ is used, upon the addition of FA cation, 2D Ruddlesden–Popper type nanostructures are always observed. Meanwhile, the weaker Lewis base R_3_P coordinated inks preferentially led to the formation of larger bulk‐like 3D perovskite FASnI_3_ NCs, attributed to their weaker adduct formation leading to weaker stabilization of the NCs. In order to fully suppress 2D nanostructure formation, an amine‐free three‐precursor approach employing zwitterionic ligands was used to synthesize stable, phase‐pure 3D FASnI_3_ NCs. With this method, the Sn, halide, and A^+^(FA/Cs) precursors are separated, allowing for more precise control over the precursor stoichiometry and reactivities.

## 2 Results and Discussion

### 2.1 SnI_2_ and its Adducts with Long‐Chain Primary Amines and Phosphines

Colloidal synthesis methods for the synthesis of NCs use coordinating ligands, such as primary amines and phosphines, to facilitate the dissolution of the SnI_2_ precursor via the formation of molecular complexes. These complexes are formed via the empty p orbital in Sn and the lone pair of electrons on the nitrogen in R‐NH_2_ and/or the phosphorus in R_3_^’^‐P ligands. Amines complex with the Sn‐nucleus to form an established adduct, with enhanced s‐p hybridization due to N electron donation, promoting the formation of species with highly symmetric environments around Sn [22]. An increase in amine concentration has shown to lead to an increase in shielding around the Sn nucleus, causing the displacement of iodine from the SnI_2_ complex, altering the ligand to metal charge transfer (LMCT) in these complexes [23]. On the other hand, substituted phosphine ligands (R_3_’‐P) change the coordination environment around Sn, but to a lower extent. The larger size and the higher polarizability of the P atom induces less hybridization, leaving the HOMO 5s^2^ electrons unaffected [24, 25]. Thus, to determine the influence of the coordination dynamics of the ligands at a molecular scale, we coupled UV–Vis absorbance of the adducts with liquid‐state ^119^Sn NMR, and complemented our experimental studies with first‐principles theoretical calculations.

**FIGURE 1:**
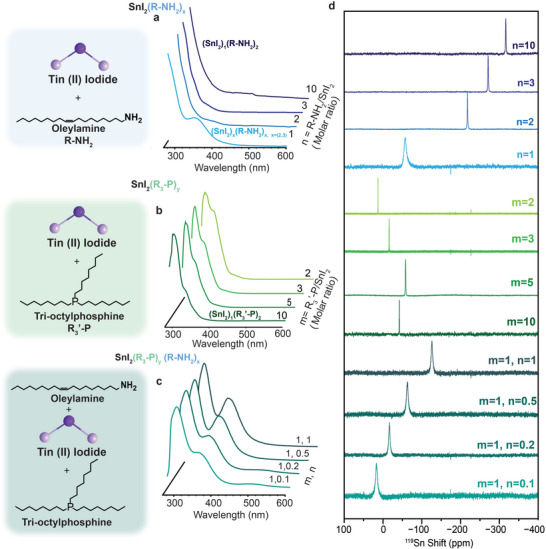
(a–c) UV–vis absorbance spectroscopy of (a) SnI_2_(R‐NH_2_)_x_, (b) SnI_2_(R’_3_‐P)_y,_ and (c) SnI_2_(R’_3_‐P)(R‐NH_2_)_x;_(d) ^119^Sn liquid‐state NMR spectroscopy of SnI_2_(R‐NH_2_)_x_, SnI_2_(R’_3_‐P)_y_ and SnI_2_(R’_3_‐P)_1_ (R‐NH_2_)x inks in toluene‐d8, where n is the molar ratio of R‐NH_2_ to SnI_2_ and m the molar ratio of R’_3_‐P to SnI_2_; x and y represent the actual numbers of coordination molecules of R‐NH_2_ and R’_3_‐P, respectively, in the formed species.

To understand the effect of the amine on tin speciation, we mixed tin iodide with R‐NH_2,_ and varied the molar ratios of Sn:ligand. At a 1:1 ratio, the resulting tin adduct showed an absorbance feature at 360 nm, which is attributed to originate from a LMCT band (Figure 1a) [26, 27]. For clarity, we denoted n as the molar ratio of R‐NH_2_ to SnI_2_ and m as the molar ratio of R’_3_‐P to SnI_2_, while x and y represent the actual numbers of coordination molecules of R‐NH_2_ and R’_3_‐P, respectively, in the formed species.

As the equivalents of R‐NH_2_ are increased (n > 2), this peak diminishes, and peaks at 282 nm become more pronounced for all equivalents (Figure S1). Coupling these observations with ^119^Sn NMR, we observed a clear trend: a broad peak at −57 ppm (n = 1, FWHM: 1100 Hz) for one equivalent of R‐NH_2_, which upon increasing equivalents evolves into sharper peaks ranging from ‐200 ppm (n = 2, FWHM: 134 Hz) to −327 ppm (n = 10, FWHM: 100 Hz), making it obvious that the adducts formed with n = 1 are distinct from the n ≥ 2 (Figure 1d) [28]. This behaviour indicates the formation of dimers or multimers in the case of low equivalents of R‐NH_2_, showing absorbance features around 330−360 nm, and significant NMR peak broadening. Upon increasing equivalents, the multimeric species may break down into monomeric adducts as shown by the loss of the distinct absorbance features and sharpening of the NMR peaks when n > 2. The presence of multimeric species in the case of n = 1 is also justified by the fast crystallization of the corresponding ink (^119^Sn shift: −57 ppm), which when dropcasted onto a silicon wafer, results in the formation of a bright red film upon contact with the substrate. Evaluation of this resulting thin film with X‐ray diffraction (XRD), revealed highly periodic peaks in the low angle regime, with a lattice spacing of ∼3.7 nm, a characteristic feature of 2D Ruddlesden–Popper (RP) type perovskite nanostructures (Figure S2a). The absorbance of this film showed a sharp peak at 577 nm, corroborating the assembly into an n = 1 RP structure after the solvent evaporation (Figure S2b). The molecular inks with n > 1 did not exhibit this behaviour, reinforcing the idea that at higher equivalents of R‐NH_2_ the multimeric species break down into smaller adducts (monomers).

In the phosphine case, when adding trioctylphosphine (R_3_’‐P) to SnI_2_, two absorbance peaks were observed at 290 nm (m = 2–5)/280 nm (m = 10) and 317 nm, irrespective of the molarity of R_3_’‐P (Figure 1b). Due to the lower Lewis basicity of R_3_‐P, dissolution of SnI_2_ was only achieved at m*> 2*, indicating that excess ligand is required to complex and stabilize the resulting tin halide adduct. The absorbance profile did not change with increasing equivalents of R_3_^’^‐P, suggesting the saturation and equilibration of the resulting SnI_2_(R_3_’‐P)_y_ complex when m = 2. In the ^119^Sn NMR spectra observed in Figure 1d, the SnI_2_(R_3_^’^‐P)_y_ complexes show sharp ^119^Sn shifts (FWHM: 60–120 Hz) ranging from 13 ppm (m = 2, FWHM: 58 Hz) to −42 ppm (m = 10, FWHM: 84 Hz). As the R_3_’‐P molarity is increased, the Sn‐nuclei become increasingly shielded, shifting upfield in increments of ∼20 ppm per equivalent, until the SnI_2_(R_3_^’^‐P)_y_ adduct saturates at m = 5. Interestingly, increasing the equivalent of R_3_’‐P further from m = 5 to m = 10 causes a downfield shift. Coupled with absorbance, this shift is also observed in the blueshift in absorbance from 290 nm (m = 2–5) to 280 nm (m = 10) (Figure 1b), indicating a structural change in the complex nature upon supersaturation with R’_3_‐P. Following this effect with ^31^P NMR, it was observed that upon complexation with Sn, the ^31^P peak of free R_3_’‐P is shifted from −32.3 ppm, to a broader multiplet at −33.6 ppm (m = 2), showcasing dynamic exchange between the bound and free R_3_’‐P (Figure S3). This peak sharpens back to a singlet for m = 3, shifting back downfield toward the free R_3_’‐P signal, indicating that the speciation of the SnI_2_(R_3_’‐P)_y_ is not dependent upon the concentration of R_3_’‐P, as in the presence of molar excess of R_3_’‐P, the system reverts back to free R_3_’‐P.

To compare the effects of resulting R‐NH_2_ and R_3_’‐P systems on the iodostannate complexes, the SnI_2_(R_3_’‐P)_y_ inks were titrated with increasing equivalents of R‐NH_2_. The absorbance profile of the resulting complexes shows two absorbance features at 295 and 360 nm (Figure 1c). The peak at 360 nm observed in Figure 1a is attributed to the presence of multimeric (SnI_2_)_x_(R‐NH_2_)_x_ as evidenced by first‐principles calculations (see below), indicating that these adducts are formed preferentially over the monomeric species. Upon increasing the eq. of R‐NH_2_, the relative ratio between the peak at 295 and 360 nm increases from 1:0.42 (m = 1, n = 0.1) to 1:0.6 (m = 1, n = 1), indicating the preferred multimer adduct formation upon addition of R‐NH_2_. NMR reinforces this observation as the ^119^Sn NMR of SnI_2_(R_3_’‐P)_1_(R‐NH_2_)_0.1_ shows the presence of one broad singular peak at 16 ppm (FWHM: 987 Hz), close to the shift of the SnI_2_(R_3_’‐P)_2_ complex (FWHM: 58 Hz) and close in broadness to the (SnI_2_)_x_(R‐NH_2_)_x_ multimeric species observed for n = 1 (FWHM: 1100 Hz). The broadening of the peak relative to the shift of the pure R_3_’‐P complex, indicates the dynamic exchange of the dimer significantly affecting the observed ^119^Sn environment of the resulting complex. The same effect is observed by ^31^P NMR (Figure S4). As R‐NH_2_ is added, the same observation of a multiplet formation was observed as that of the SnI_2_(R_3_’‐P)_y_ system, with the splitting into a doublet at m = 0.5 with a J‐coupling of ∼8 Hz, indicative of ^31^P–^1^H coupling, indicating dynamic exchange (Figure S4) [29]. Addition of further equivalents of amine results in a singlet peak shifting the ^31^P signal downfield toward its original position, suggesting that the amine substitution is complete. The reverse titration on the other hand, via the titration of R_3_’‐P into the SnI_2_(R‐NH_2_)_1_ solution, causes an upfield shift by ∼50 ppm for initial R_3_’‐P equivalents and saturates thereafter, indicating complete coordination with Sn (Figure S5). With increasing equivalents of R_3_’‐P, the Sn signal shifts downfield by only ∼10 ppm, again reinforcing the notion of the preferential binding of Sn to R‐NH_2_ over R_3_^’^‐P.

To better understand the structure of the adducts formed in all three cases, we investigated their nature using density functional theory (DFT) calculations (see the Supporting Information for full computational details) [30]. Monomeric SnI_2_(R‐NH_2_)_x_, SnI_2_(R_3_’‐P)_y,_ Sn(R‐NH_2_)_x_^2+^, Sn(R_3_’‐P)_y_^2+^, and multimeric (SnI_2_)_x_(R‐NH_2_)_x_ (x = 2−4) and (SnI_2_)_2_(R_3_’‐P)_2_ were fully characterized at the M062X‐D3/def2‐TZVP+CPCM(Toluene) level of theory [31, 32, 33, 34]. The stability of the resulting adducts was rationalized by the interaction energy between the ligands and the precursors (*E*_int_), and by the Sn─P and Sn─N bond strength as evaluated through the intrinsic bond strength index (IBSI) [35]. Theoretical calculations indicate that both R‐NH_2_ and R_3_’‐P strongly bind SnI_2_, with *E*_int_ of −31.3 and −37.0 kcal/mol, respectively, to form SnI_2_(R‐NH_2_/R_3_’‐P)_x/y_ with x/y = 1 (Figure S6), whereas the slightly larger IBSI calculated for the R‐NH_2_ complex suggests that the Sn‐N local coordination is stronger compared to Sn‐P for R_3_’‐P (Tables S1 and S2). The stronger coordination of the R‐NH_2_ ligand was also confirmed by the larger IBSI value for the Sn‐N bond (0.104) in the SnI_2_(R‐NH_2_)(R_3_’‐P) adduct (Figure S7, x:y 1:1) compared to that for the Sn─P bond of the R_3_’‐P ligand (0.099). Noteworthy, insertion of additional ligands in x,y = 2 and 3 leads to a decrease in the interaction energy, especially for R_3_’‐P due to its less stabilizing interaction between the aliphatic chains (Figure S6). Again, the IBSI values indicate that R‐NH_2_ is coordinated stronger than R_3_’‐P for x,y = 2 and 3 (Tables S1 and S2). SnI_2_(R‐NH_2_)_x_ and SnI_2_(R_3_’‐P)_y_ with x,y = 4 were predicted unstable, with one of the ligands detaching from the coordination. The presence of Sn(R‐NH_2_)_x_^2+^ and Sn(R_3_’‐P)_y_^2+^ adducts in solution was also discarded as the IBSI values for the Sn─N and Sn─P bonds are significantly smaller than those calculated for Sn─I in SnI_2_ (Table S3). Dimeric (SnI_2_)_2_(R‐NH_2_)_2x_ and (SnI_2_)_2_(R_3_’‐P)_2y_ adducts were modelled, but only the (SnI_2_)_2_(R‐NH_2_)_2_ (Figure S8) was calculated relatively stable: 3.17 and 6.68 kcal/mol per monomer unit higher in energy than monomeric SnI_2_(R‐NH_2_) when iodine atoms are in *trans* and *cis* conformation, respectively. Both species might therefore be present experimentally at low concentrations of R‐NH_2_. Increasing the coordination to x = 2 as in (SnI_2_)_2_(R‐NH_2_)_4_, or moving to the R_3_’‐P analogue of (SnI_2_)_2_(R_3_’‐P)_2_, lead to the disruption of the complex into their monomer counterparts. Finally, trimeric and tetrameric (SnI_2_)_x_(R‐NH_2_)_x_ with x = 3−4 were modelled. Theoretical calculations indicate that (SnI_2_)_3_(R‐NH_2_)_3_ trimer (Figure S9) is even more stable than the monomer species by −7.32 kcal/mol/unit, whereas the (SnI_2_)_4_(R‐NH_2_)_4_ tetramer decomposes into trimer and monomer fragments.

Time‐dependent DFT (TD‐DFT) calculations were performed for the different R‐NH_2_ and R_3_’‐P adducts to investigate their UV–vis absorption features. All the monomeric SnI_2_(R‐NH_2_/R_3_’‐P)_x/y_ species with x/y = 1–3 display an intense band below 300 nm, which correlates with the experimental peak recorded around 300 nm for all the inks (compare Figure 1 with Figure S10 for the R‐NH_2_‐based adducts and Figure S11 for the R_3_’‐P‐based adducts and the SnI_2_(R‐NH_2_)(R_3_’‐P) complex). The main electronic transition responsible for that band, similar for both types of adducts, is mainly centered on the SnI_2_ core (see Figure S12 for SnI_2_(R‐NH_2_)). For both R_3_’‐P‐based adducts and the SnI_2_(R‐NH_2_)(R_3_’‐P) complex, similar absorption spectra are predicted, with an intense band around 330 nm with the same nature (Figure S13). The absorption feature recorded experimentally near 360 nm for SnI_2_(R‐NH_2_)_x_ (n = 1) could be explained by the multimer (SnI_2_)_x_(R‐NH_2_)_x_ (x = 2−3) adduct, which is predicted with intense electronic transitions arising from iodine induced ligand‐to‐metal charge transfer (LMCT) in the range of 350–370 nm for the dimer and 320–330 nm for the trimer (Figures S14 and S15, respectively). By increasing the SnI_2_:R‐NH_2_ ratio (n > 1), adducts of the type (SnI_2_)_2_(R‐NH_2_)_x_ (x > 2) decompose into their SnI_2_(R‐NH_2_)_x_ counterparts, explaining the experimental disappearance of that absorption feature. Based on the interaction energy and bond strength analysis, combined with the absorption features of the different species, we suggest that upon increasing the R‐NH_2_ content, an initial multimeric SnI_x_(R‐NH_2_)_x_ (x = 2−3) species evolves to monomeric SnI_2_(R‐NH_2_)_x_ with x ≤ 3, as supported by the NMR experiments. In contrast, R_3_’‐P insertion directly leads to the monomeric species SnI_2_(R_3_’‐P), which will hardly incorporate additional ligands to the coordination.

### 2.2 From SnI_2_ Adducts to FASnI_3_ Nanostructures

**FIGURE 2:**
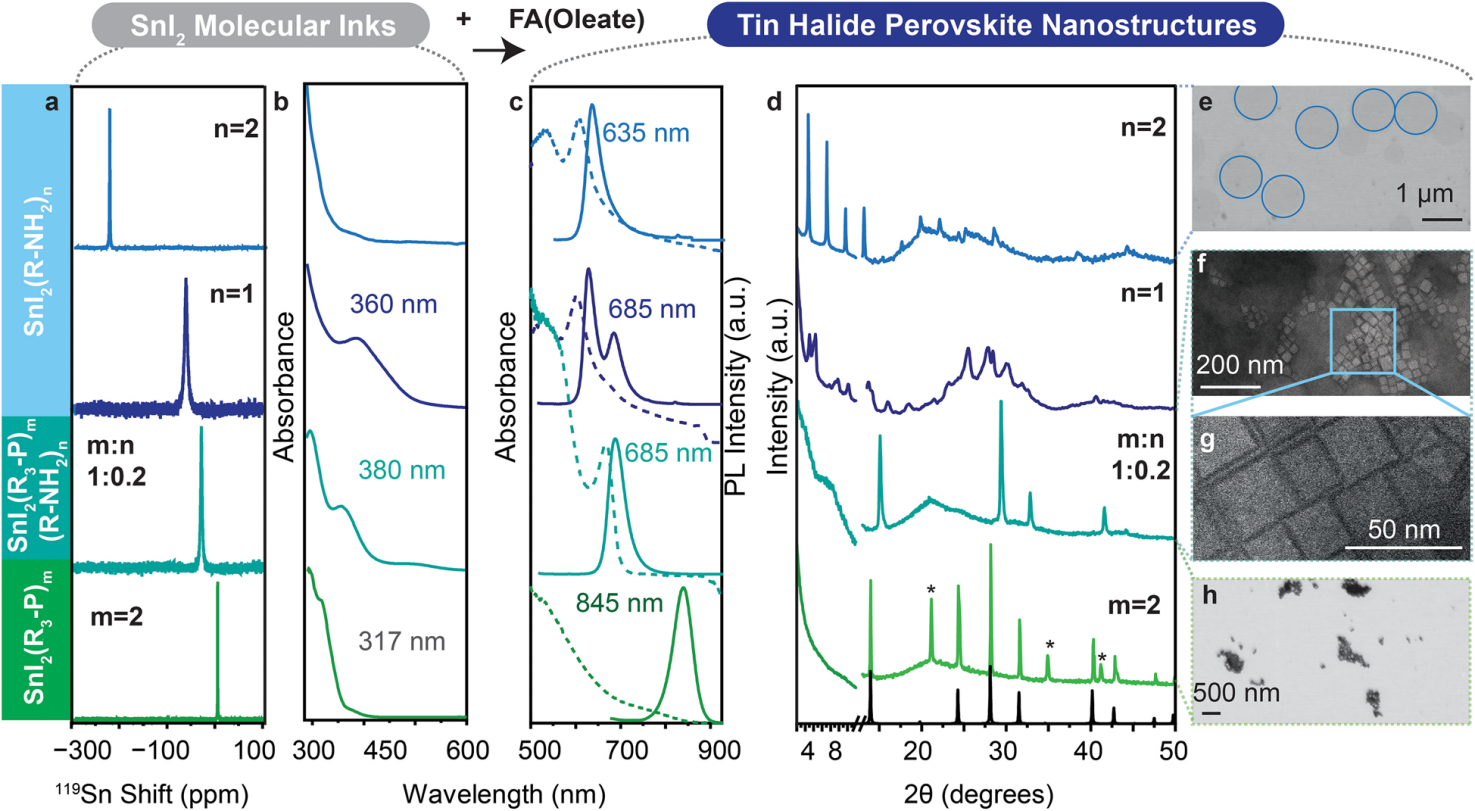
(a) ^119^Sn spectra of SnI_2_(R‐NH_2_)_x_ complexes, SnI_2_(R_3_’‐P)_1_(R‐NH_2_)_0.2_ and SnI_2_(R_3_^’^‐P)_2_ complex, (b) UV–vis absorbance spectra of SnI_2_(R‐NH_2_)_m_ complexes, SnI_2_(R_3_^’^‐P)_1_(R‐NH_2_)_0.2_ and SnI_2_(R_3_’‐P)_2_ complex, (c) UV–vis absorbance spectra (dotted line) and PL spectra of FASnI_3_ nanostructures formed after injection of FA‐oleate into SnI_2_(R‐NH_2_)_m_ complexes, SnI_2_(R_3_’‐P)_1_(R‐NH_2_)_0.2_ and SnI_2_(R_3_’‐P)_2_ complex, (d) XRD diffractograms of FASnI_3_ nanostructures formed after injection of FA‐oleate into SnI_2_(R‐NH_2_)_x_ complexes, SnI_2_(R_3_^’^‐P)_1_(R‐NH_2_)_0.2_ and SnI_2_ (R_3_^’^‐P)_2_ complex, (e) SEM image of nanosheets formed from the SnI_2_(R‐NH_2_)_2_ complex, (f) STEM image of FASnI_3_ formed from SnI_2_(R_3_^’^‐P)_1_(R‐NH_2_)_0.2,_ (g) zoomed in STEM image of of FASnI_3_ formed from SnI_2_(R_3_^’^‐P)_1_(R‐NH_2_)_0.2,_(h) SEM of bulk FASnI_3_ obtained from the SnI_2_(R_3_^’^‐P)_2_ ink.

With these insights into the molecular nature of the tin iodide complexes with R‐NH_2_ and R_3_’‐P, the next step was to test how this directly translates into the formation of FASnI_3_ nanostructures. Formamidinium oleate (FA‐Oleate) was injected into these inks (SnI_2_ complexes with our Lewis bases at various ratios) at room temperature, resulting in the rapid formation of FASnI_3_ nanostructures. Post FA‐oleate injection, featureless SnI_2_(R‐NH_2_)_2_ complex (Figure 2a,b (light blue)), evolves to show sharp absorbance and photoluminescence (PL) at 635 nm (Figure 2c (light blue)). The low angle peaks in XRD, with lattice spacing of 3.9 nm, showcase the formation of a 2D RP type nanostructure. SEM reinforces this observation, with the presence of large nanosheets observed analogous to RP nanostructures (Figure 2e). After injecting FA‐oleate into the (SnI_2_)_x_(R‐NH_2_)_x_ multimer (x > 1), the absorbance feature of at 360 nm (Figure 2b (dark blue)) shifts to smaller energies (Figure 2c (dark blue)), which via XRD was shown to correspond to a mixture of 2D RP structures and FASnI_3_ NCs (Figure 2d (dark blue)). The presence of the double PL peaks at 635 and 685 nm indicate the presence of two PL active nanostructures (Figure 2c (dark blue)).

On the other hand, when FA‐oleate is injected into the mixed ligand SnI_2_(R_3_^’^‐P)_1_(R‐NH_3_)_0.2_ complex_,_ one singular PL peak at 685 nm (Figure 2c (teal)) is observed, indicating primarily that one type of nanostructure is present. XRD corroborated this observation where the diffractogram corresponded to pure FASnI_3_, without strong evidence of 2D nanostructures in the low angle regime (Figure 2d (teal)). High‐resolution scanning transmission electron microscopy (HR‐STEM) revealed the presence of cubic NCs with a size of 23 ± 5 nm (Figure 2f). Upon closer look, between some of the NCs, a linear type of structure was observed, which is attributed to the formation of nanoribbons between the FASnI_3_ NCs (Figure 2g) [36]. This indicates that in the presence of even minimal amounts of multimeric species, the formation of 2D type nanoribbons is preferred. This preferential stacking into nanoribbons was also observed by Mitzi et al. where SnI_2_ was reacted to dodecylammonium iodide leading to nanoribbons of the [SnI_3_^−^] framework analogous to the MoO_3_ framework. [36]

When using inks utilizing R_3_’‐P as the sole ligand, the formation of NCs with a PL peak at 845 nm (Figure 2c (green)) was observed, corresponding to pure 3D FASnI_3_ large structures. The absence of features in the low‐angle regime excludes the presence of 2D nanostructures (Figure 2d (green)). The presence of a crystalline side‐product, not corresponding to any known Sn nanostructure, was observed besides the obtained FASnI_3_ NCs (Figure 2d* (green)). SEM showed that the weakly binding nature of R’_3_‐P was unable to fully stabilize the NCs, with rapid growth causing the aggregation into bulk‐like materials (Figure 2h). Increasing the equivalents of R_3_^’^‐P did not result in better stabilization of the NCs, with the formation of phase pure bulk‐like NCs observed when molarity is varied from m = 2 to 4 (Figure S16). This aggregation into larger clusters can also be seen from the absorbance spectra of these NCs, where a broad, featureless spectrum indicates a large size distribution as well as poorly defined excitonic features (Figure 2c (green)). It is also important to note that all the resulting nanostructures show low photoluminescence quantum yield (PLQY) with values in the range of 0%–1%. Using these insights, we can rationalize that, even with minimal amounts of amine present, the stronger binding to Sn favours the formation of 2D nanostructures. In the absence of an amine, when R_3_’‐P is used, the binding strength of this ligand is insufficient to stabilize the resulting nanoparticles on its own, resulting in the formation of large bulk‐type FASnI_3_.

### 2.3 Amine‐Free Synthesis of FASnI_3_ NCs

**FIGURE 3:**
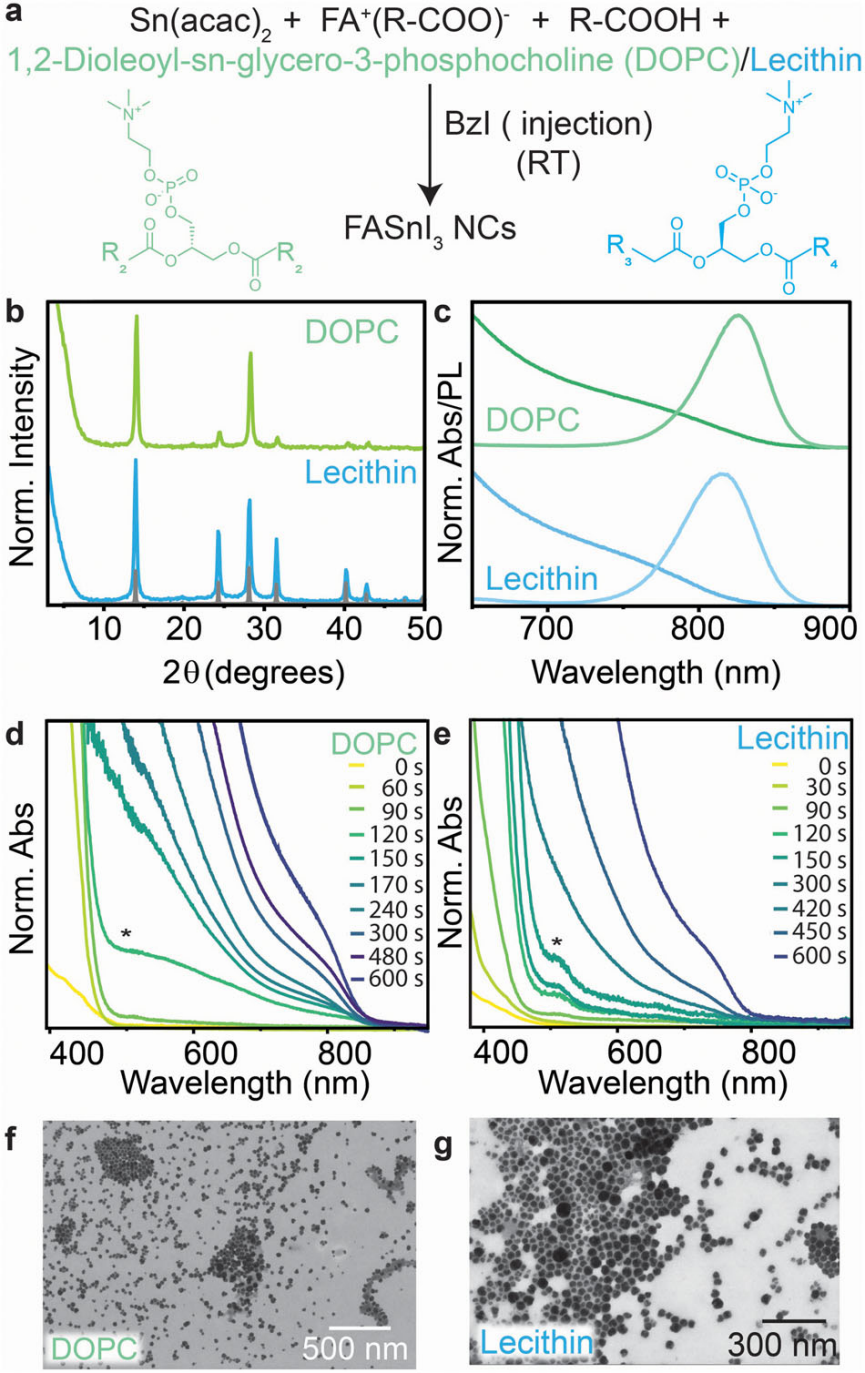
(a) Reaction scheme for amine‐free three precursor approach of FASnI_3_ NCs using 1,2‐Dioleyl‐sn‐glycero‐3‐phosphocholine (DOPC) R_2_:C_17_H_33_, and Lecithin R_3_:C_17_H_31_, R_4_:C_15_H_31_, (b) XRD of DOPC and Lecithin capped FASnI_3_ NCs, (b) Normalized absorbance and PL of DOPC and Lecithin capped FASnI_3_ NCs, (d) Insitu absorbance of DOPC capped FASnI_3_ over the range of 600s, (e) In situ absorbance of Lecithin capped FASnI_3_ over the range of 600s, (f) STEM image of DOPC capped FASnI_3_ NCs, (g) STEM image of Lecithin capped FASnI_3_ NCs.

We concluded in the previous sections that, in order to synthesize stable pure 3D FASnI_3_ and avoid the formation of unwanted 2D nanostructures NCs, an amine free approach is required. Moreover, a stronger binding ligand than R_3_’‐P is also required to stabilize the resulting NCs and reduce the surface energy to prevent it from growing into bulk, as seen in the case of pure R_3_’‐P. To control this and to obtain stable and phase pure 3D NCs, we used an amine‐free three‐precursor approach incorporating the stronger binding zwitterionic capping ligand lecithin as the primary capping ligand. [37, 38, 39, 40, 41, 42] One disadvantage of employing the lecithin precursor, however, is the lack of control over the composition of fatty acids present in the compound. Lecithin is a general term defining a mixture of phospholipids, hence the precise stoichiometry of the ligand in the reaction is difficult to determine. Thus, to have precise control over the stoichiometry of the zwitterionic ligand used, this method was extended to the use of a phosphocholine, 1,2‐dioleyl‐sn‐glycero‐3‐phosphocholine (DOPC) as the zwitterionic capping ligand [42, 43]. This ligand was also chosen due to its long‐chain oleyl moieties, analogous to R‐NH_2_. Furthermore, in order to achieve better control over the nucleation and growth of the NCs, we separated the halide from the tin by employing tin acetylacetonate (Sn(acac)_2_) and benzoyl iodide (BzI) as the respective sources (Figure 3a) [41]. This separation ensures better control over precursor speciation in the ink, thereby governing the nucleation and growth of the NCs. Additionally, the high reactivity of these precursors allows for the room‐temperature synthesis of these NCs, allowing for the in situ characterization of the growth process.

With this method, pure 3D FASnI_3_ NCs were obtained within 10 min, with XRD confirming the absence of 2D nanostructures via a featureless low‐angle regime (Figure 3b). The resulting NCs show sharp PL at 825 nm (DOPC) and 815 nm (Lecithin) and a defined broad absorption feature around 800 nm for both systems (Figure 3c). The formation of the NCs was followed by in situ absorbance, revealing that the growth mechanism proceeds via the formation of intermediate clusters at 500–520 nm (marked with an ^*^) (Figure 3d,e). Similar observations were reported for Pb halide perovskite synthesis when benzoyl halides were employed [44]. We noted minor structural differences between the two zwitterions resulted in different nucleation and growth mechanisms. Faster growth was observed for the DOPC ligand, with the growth of the NCs beginning around 120 s (Figure 3d*) whereas for the lecithin, the growth from the intermediate clusters occurs more slowly after 300 s (Figure 3e*). It has been shown that lecithin shows strong and static binding to the surfaces of NCs, however the diffusion of free lecithin in solution is low, thereby delaying the nucleation and growth [45]. SEM imaging showed the formation of quasi‐spherical NCs with broad size distributions with an average size 30 ± 5 nm for the DOPC capped NCs, while the lecithin capped NCs showed an average size of 32 ± 8 nm. (Figure 3f,g). [46] It is important to note that the fine control over the growth process cannot be achieved yet, resulting in a broad size distribution as well as slight differences in size distributions from batch to batch (Figure S17).

While the preferential formation of 2D nanostructures in the presence of amines results in NCs with reduced colloidal stabilities (∼24–48 h), the NCs formed with our amine‐free method both show longer optical and colloidal stabilities (∼1 month), highlighting the efficacy of these zwitterionic ligands for the formation of phase pure 3D tin halide perovskites.

## 3 Conclusion

In this work, we systematically investigated the complexation dynamics of the tin precursor SnI_2_ with benchmark Lewis base ligands, amines (R–NH_2_) and phosphines (R′_3_–P), using correlated in situ NMR and UV–vis spectroscopies supported by theoretical calculations. Our results establish that both ligand families form stable monomeric SnI_2_ adducts, yet amines consistently exhibit stronger Sn─N coordination than the Sn─P bonding observed for phosphines. Computational analysis further revealed that higher ligand loadings destabilize these complexes, particularly for phosphines. On the other hand, only R‐NH_2_‐based multimeric (SnI_2_)_x_(R‐NH_2_)_x_ (x = 2−3) species are predicted relatively stable, which are expected to be present under low ligand conditions. TD‐DFT calculations clarified the origin of key absorption features, and confirmed that the multimeric species progressively evolve into monomeric SnI_2_(R–NH_2_)_x_ adducts (x ≤ 3) as the amine concentration increases. Crucially, these molecular insights directly correlate with the dimensionality of FASnI_3_ nanostructures formed from the corresponding inks. Stronger Lewis bases such as amines consistently steer the system toward 2D Ruddlesden–Popper phases, even at low concentrations, while their strong binding simultaneously stabilizes the resulting NCs. In contrast, weaker phosphine coordination leads preferentially to the formation of larger, bulk‐like 3D perovskite FASnI_3_ NCs, a consequence of weaker adduct formation and insufficient stabilization, which promote rapid growth and aggregation. Building on these findings, we used an amine‐free three‐precursor strategy that suppresses unwanted 2D phases and enables the synthesis of phase pure 3D FASnI_3_ NCs. This approach employs a strongly binding zwitterionic ligand (such as DOPC or lecithin) and separates the Sn and halide sources (Sn(acac)_2_ and BzI), providing enhanced control over stoichiometry and precursor reactivity. The resulting NCs exhibit markedly improved optical and colloidal stability (∼1 month) compared to the short lifetimes (∼24–48 h) observed when amines are present. Overall, the fundamental understanding gained here regarding SnI_2_ coordination chemistry establishes a solid foundation for the rational design of more predictable and robust molecular inks. This mechanistic insight will be instrumental in disentangling the competing pathways that govern phase selectivity and dimensionality, ultimately accelerating the development of tin halide perovskite and perovskitoid nanostructures with targeted properties.

## Conflicts of Interest

The authors declare no conflicts of interest.

## Supporting information




**Supporting File**: smll72745‐sup‐0001‐SuppMat.docx.

## Data Availability

The data that support the findings of this study are available from the corresponding author upon reasonable request.
