# Supplementary material for: The Chemistry From Tin Iodide Molecular Inks to FASnI3 Nanocrystals
Source: Small. 2026 Feb 6;22(20):e11842. doi: 10.1002/smll.202511842 (PMC13054445; doi:10.1002/smll.202511842)
Supplement: Supplementary file 1 — Supporting File: smll72745‐sup‐0001‐SuppMat.docx. [file SMLL-22-e11842-s001.docx]

Supporting Information

The Chemistry from Tin Iodide Molecular Inks to FASnI₃ Nanocrystals

Kushagra Gahlot^┼[a]^, Julia N. Kraft^┼[a]^, Manuel Pérez-Escribano^[b]^, Mihai T. Todosia^[a]^, Karla Ravin^[a]^_,_ Joaquín Calbo^[b]^ and Loredana Protesescu^[a]^*

^a^Zernike Institute for Advanced Materials, University of Groningen, Nijenborgh 3, Groningen, 9747AG, The Netherlands. E-mail: [l.protesescu@rug.nl](mailto:l.protesescu@rug.nl)

^b^Instituto de Ciencia Molecular, Universitat de València, c/Catedrático José Beltrán, 2, 46980 Paterna, Spain

**Table of Contents**

|  | Description | Page |
| --- | --- | --- |
| **I** | Experimental section | 3-7 |
| **II** | Characterization techniques | 8 |
| **III** | Supporting Figures | 9-16 |
| **IV** | References | 17 |
|  | | |
| **Figure S1** | Absorbance of SnI_2_(R-NH_2_)_n_ complexes, expanded region to 270 nm. | 9 |
| **Figure S2** | XRD pattern UV-visible spectroscopy of SnI_2_: OLA = 1: 1 yellow solution drop-casted on a Si wafer. | 9 |
| **Figure S3** | ^31^P NMR for SnI_2_(R’_3_-P)_m_ solutions. | 10 |
| **Figure S4** | ^31^P NMR spectra for SnI_2_(R’_3_-P)_1_(R-NH_2_)_x_ inks. | 10 |
| **Figure S5** | ^119^Sn NMR spectra for R_3_-P titration in the different SnI_2_( R-NH_2_)_1_ inks. | 11 |
| **Figure S6** | M062X/def2-TZVP+CPCM(Toluene) minimum-energy structures of the SnI_2_(R-NH_2_)_x_ complexes and SnI_2_(R_3_’-P)_x_ complexes obtained from the iterative GFN2-xTB docking procedure. | 11 |
| **Figure S7** | M062X/def2-TZVP+CPCM(Toluene) minimum-energy structure of the SnI_2_(R-NH_2_)(R_3_’-P) adduct. | 12 |
| **Table S1** | M062X/def2-TZVP+CPCM(Toluene) SnI_2_(R-NH_2_)_x-1_-R-NH_2_ interaction energy (E_int_) and Sn-Nand Sn-I IBSI values. | 12 |
| **Table S2** | M062X/def2-TZVP+CPCM(Toluene) SnI_2_(R_3_’-P)_x-1_-R_3_’-P interaction energy (E_int_) and Sn-P and Sn-I IBSI values. | 12 |
| **Table S3** | M062X/def2-TZVP+CPCM(Toluene) Sn-N and Sn-P IBSI values for the Sn(R-NH_2_/R_3_’-P)_x_^2+^ (x=1 and x=2, respectively) adducts and Sn-I IBSI values for the molecular SnI_2_. | 12 |
| **Figure S8** | M062X/def2-TZVP+CPCM(Toluene) minimum-energy structure of the (SnI_2_)_2_(R-NH_2_)_2_ adducts: cis (left), trans (right) | 13 |
| **Figure S9** | M062X/def2-TZVP+CPCM(Toluene) minimum-energy structure of the (SnI_2_)_3_(R-NH_2_)_3_ adduct | 13 |
| **Figure S10** | B3LYP-D3/def2-TZVP+CPCM (Toluene) predicted absorption spectra for the different R-NH_2_-based adducts. | 13 |
| **Figure S11** | B3LYP-D3/def2-TZVP+CPCM (Toluene) predicted absorption spectra for the different R_3_’-P-based adducts. | 14 |
| **Figure S12** | B3LYP-D3/def2-TZVP+CPCM (Toluene) natural transition orbitals (NTOs) involved in the most intense electronic transition around 300 nm for both SnI_2_(R-NH_2_) and SnI_2_(R_3_’-P) | 14 |
| **Figure S13** | B3LYP-D3/def2-TZVP+CPCM (Toluene) natural transition orbitals (NTOs) involved in the most intense electronic transition around 330 nm both SnI_2_(R_3_’-P) and SnI_2_(R-NH_2_)(R_3_’-P). | 14 |
| **Figure S14** | B3LYP-D3/def2-TZVP+CPCM (Toluene) natural transition orbitals (NTOs) involved in the most intense electronic transitions in the 300-400 nm range for the SnI_2_(R-NH_2_)_2_ trans dimer. | 15 |
| **Figure S15** | *B3LYP-D3/def2-TZVP+CPCM (Toluene) natural transition orbitals (NTOs) involved in the most intense electronic transitions in the 300-400 nm range for the (SnI_2_)_3_(R-NH_2_)_3_ trimer* | 15 |
| **Figure S16** | XRD of FASnI_3_ formed in various SnI_2_(R_3_-P)_y_ inks. | 16 |
| **Figure S17** | Size distribution for a) DOPC and b) Lecithin capped FASnI_3_ NCs. | 16 |

**I. Experimental Section**

**Materials:**

Formamidine acetate (FAAc, > 98%, TCI Europe), tin (II) iodide (Sigma, anhydrous beads, 99.99%), tri-octyl phosphine (R’_3_-P, 97%, Sigma), oleyl amine (R-NH_2_, tech. grade, 70%, Sigma), oleic acid (OA, tech. grade, 90%, Sigma), octadecene (ODE, tech. grade, 90%, Sigma), toluene (Arcos, extra dry, 99.85%), toluene-d8 (≥ 99.0%, Sigma), tin (II) acetyl acetonate (Sn(acac)_2_, > 99%, ABCR), benzoyl chloride (BzCl, 99%, Sigma), Sodium iodide (NaI, 99.999%, Sigma), lecithin (> 97%, biochem. grade, Roth), 1,2-dioleoyl-sn-glycero-3-phosphocholine (DOPC, >97.0%, TCI Europe). R’_3_-P, R-NH_2,_ OA, and ODE were dried under high vacuum for 3 hrs at 105⁰C and transferred to glove box carefully for further use.

**Preparation of NMR solutions**

**SnI_2_(R-NH_2_)_x_ solutions**

SnI_2_ (0.25 mmol, 0.0935 g) was mixed with R-NH_2_ and toluene-d8 in a 4 ml glass vial equipped with a magnetic stirring bar. This solution was kept for stirring at room temperature to obtain transparent yellow solutions. SnI_2_ salt dissolves completely if the molar concentration of R-NH_2_ is equal or more than 1 with respect to Sn.

| **SnI_2_: R-NH_2_ Ratio** | **SnI_2_ (g)** | **R-NH_2_ (μL)** | **Tol-d (μL)** | **Total Volume (μL)** |
| --- | --- | --- | --- | --- |
| 1: 1 | 0.0935 | 82.5 | 600 | 682.5 |
| 1: 2 | 0.0935 | 165 | 517.5 | 682.5 |
| 1: 3 | 0.0935 | 247.5 | 435 | 682.5 |
| 1: 5 | 0.0935 | 412.5 | 270 | 682.5 |
| 1: 10 | 0.0374 | 330 | 352.5 | 682.5 |

**SnI_2_(R’_3_-P)_y_ solutions**

SnI_2_ (0.25 mmol, 0.0935 g) was mixed with R’_3_-P and toluene in a 4 ml glass vial with magnetic stirring bar. This solution was kept for stirring at the room temperature to obtain transparent yellow solutions. SnI_2_ salt dissolves completely if the molar concentration of R’_3_-P is equal or more than 2 with respect to Sn.

| **SnI_2_: R’_3_-P Ratio** | **SnI_2_ (g)** | **R’_3_-P (μL)** | **Tol-d (μL)** | **Total Volume (μL)** |
| --- | --- | --- | --- | --- |
| 1: 2 | 0.0935 | 225 | 487.5 | 712.5 |
| 1: 3 | 0.0935 | 347.5 | 375 | 712.5 |
| 1: 5 | 0.0935 | 562.5 | 150 | 712.5 |
| 1: 10 | 0.0075 | 225 | 487.5 | 712.5 |
| 1: 20 | 0.0075 | 450 | 262.5 | 712.5 |

**SnI_2_(R’_3_-P)_y_(R-NH_2_)_x_ titration experiments**

**R’_3_-P titrations -** SnI_2_(R-NH_2_)_x_ (x = 1, 2) NMR solutions were prepared. For each equivalent addition, 112.5 μL of R’_3_-P was added.

**R-NH_2_ titrations -** SnI_2_(R’_3_-P)_2_ and SnI_2_(R’_3_-P)_1_(R-NH_2_)_0.1_ NMR solutions were prepared. For each equivalent addition, 82.5 μL of R-NH_2_ was added.

**FA(Ol) injection for the Synthesis of FASnI_3_ NCs**

**Synthesis of 0.25 M FA(Ol) -** Formamidinium acetate (0.26 g, 2.5 mmol), OlAc (1 ml, 3 mmol), and ODE (9 ml) were loaded into a 25 ml 3-neck round-bottom flask and vigorously stirred under vacuum for one hour at 50 °C and then temperature is increased slowly to 105c°C. The reaction mixture was degassed for an hour. Now under N_2_ flow, the reaction mixture was heated up to 160 °C to assure the complete conversion of formamidine acetate salt into formamidinium oleate (FA(Ol)). The obtained FA(Ol) solution was cooled to room temperature and subsequently transferred to the glove box.

The above NMR solutions were utilized as a precursor/pre-injection solution. FA(Ol) was injected in a vigorously stirring precursor solution. After 10s, the flask was removed from stirring and the reaction mixture was transferred to a centrifuge tube for the purification step.

1. ***SnI_2_(R’_3_-P)_y_ solutions:*** 400 μL of FA(Ol) solution was injected. For purification, the reaction mixture was centrifuged for 15k rpm for 8 minutes. The translucent grey supernatant was discarded and precipitate was redissolved in 1 mL toluene. This solution was centrifuged further at 8k rpm for 5 minutes. The transparent supernatant was discarded and the precipitate was redissolved in 1 mL toluene, collected for further characterization. Inferior colloidal stability was observed for the solution.
2. ***SnI_2_(R-NH_2_)_x_ solutions:*** For SnI_2_: R-NH_2_ = 1: 1, 400 μL of FA(Ol) solution was injected. For purification, the reaction mixture was centrifuged for 15k rpm for 8 minutes. The translucent grey supernatant was discarded and precipitate was redissolved in 1mL toluene. This solution was centrifuged further at 5k rpm for 5 minutes. The transparent supernatant and the precipitate were collected for further characterization. For SnI_2_: R-NH_2_ = 1: 2, after injection of 400 μL of FA(Ol) solution – no stable structures were observed as they formed over injection and redissolved again in the solution. Stable nanostructures were observed on an injection of >1200 μL of FA(Ol) solution. The clear transparent supernatant was discarded and precipitate was redissolved in 1 mL toluene. This solution was centrifuged further at 5k rpm for 5 minutes. Clear supernatant was again discarded and deep red precipitate was collected for further characterization.
3. ***SnI_2_(R’_3_-P)_y_(R-NH_2_)_x_ solutions:*** 400 μL of FA(Ol) solution was injected. For purification, the reaction mixture was centrifuged for 15k rpm for 8 minutes. The translucent grey supernatant was discarded and precipitate was redissolved in 1mL toluene. This solution was centrifuged further at 5k rpm for 5 minutes. The precipitate was discarded and the supernatant was carefully collected for further characterization. The supernatant can be further purified the next day to get rid of aggregates by centrifugation at 3k rpm for 5 minutes and carefully collecting the supernatant.

**Amine-free Synthesis of FASnI_3_ NCs**

**Synthesis of Benzoyl Iodide (BzI) -** The benzoyl iodide was synthesized following the modified procedure from Theobald and Smith.^1^ In the glove box, benzoyl chloride (2.8 mL) was added to a 20 mL glass vial containing 6 g of sodium iodide. This reaction mixture was vigorously stirred overnight at 80⁰C for 6 hrs. The dark orange red solution was diluted with 3 mL toluene and filtered with 0.45 μm PTFE filter. This solution was then utilized for injection.

**Lecithin solution –** 1 g of lecithin was dissolved 5 mL of toluene. Solution can be mildly heated for complete dissolution.

**Lecithin capped FASnI_3_ synthesis -** In a glovebox, Sn(acac)_2_ (48 μL, 0.22 mmol, 1 eq.), FA(Ol) 0.5 M (400 μL, 0.22 mmol, 1 eq.), OA (127 μL, 0.44 mmol, 2 eq.), lecithin solution (400 μL) and toluene (3 mL) were stirred together in a 20 mL vial until homogeneous. At room temperature, BzI (180 μL, 0.66 mmol, 3 eq.) was injected using a 1 mL pipette under vigorous stirring. Over 5 minutes, the solution turns from yellow to dark red. The reaction was allowed to stir for 10 minutes total. For purification, the crude was equally divided in two centrifuge tubes and centrifuged at 13400 rpm for 5 minutes. The supernatant was discarded, and the precipitate redispersed in toluene (1 mL), followed by centrifugation at 5000 rpm for 5 minutes. The precipitate was discarded, and the supernatant was centrifuged at 13400 rpm for 3 minutes. The supernatant was discarded, the precipitate was redispersed in toluene (1 mL), and centrifuged at 5000 rpm for 3 minutes. The precipitate was discarded, and the supernatant was centrifuged once more at 13400 rpm for 3 minutes. The supernatant was discarded, and the precipitate was redispersed in toluene (1 mL) to obtain the product as a colloidally stable, dark red compound.

The synthesis was attempted by using only OA or only lecithin as the available capping ligands. However, this lead to the formation of non-soluble, bulk product. Different molar ratios of Sn:FA were also tried (from Sn:FA = 1:0.625 to Sn:FA = 1:2), but ratios other than Sn:FA = 1:1 did not lead to stable compound.

**DOPC zwitterion capped FASnI_3_ synthesis –** this section discusses a synthetic approach in which lecithin was replaced by DOPC 0.1 M in toluene as the capping zwitterion

In a glovebox, Sn(acac)_2_ (48 μL, 0.22 mmol, 1 eq.), FA(Ol) 0.5 M (400 μL, 0.22 mmol, 1 eq.), OA (127 μL, 0.44 mmol, 2 eq.), DOPC 0.1 M in toluene (330 μL, 0.033 mmol, 0.15 eq.) and toluene (3 mL) were stirred together in a 20 mL vial until homogeneous. At room temperature, BzI (180 μL, 0.66 mmol, 3 eq.) was injected using a 1 mL pipette under vigorous stirring. Over 5 minutes, the solution turns from yellow to dark red. The reaction was allowed to stir for 10 minutes total. For purification, the crude was equally divided in two centrifuge tubes and centrifuged at 13400 rpm for 5 minutes. The supernatant was discarded, and the precipitate redispersed in toluene (1 mL), followed by centrifugation at 5000 rpm for 5 minutes. The precipitate was discarded, and the supernatant was centrifuged at 13400 rpm for 3 minutes. The supernatant was discarded, the precipitate was redispersed in toluene (1 mL) to obtain the product as a colloidally stable, dark red compound.

The synthesis was also attempted by increasing the ratio of DOPC to Sn. It was found that a higher amount of DOPC than the molar ratio Sn:DOPC = 1:0.5 does not give product, as it seems Sn binds very strongly to the DOPC moiety.

**II. Characterization techniques**

**UV-Visible absorbance spectroscopy.** The absorption measurements were performed on a Table-top Avantes UV-Vis spectrophotometer using a tungsten and halogen filament lamp as the excitation source (in the glove box). In-situ absorbance measurements were performed by aquiring the absorbance spectra every 30s. Absorption spectra were normalized using the excitonic peak, defined as the maximum intensity at the corresponding wavelength.

**Steady-state PL spectroscopy.** The photoluminescence measurements were performed on a Horiba Scientific Jobin Yvon spectrometer equipped with a PMT detector.

**XRD.** Powder X-ray diffraction measurements were performed in inert dome sample holder in a Bruker D8 Advanced diffractometer in Bragg-Brentano geometry using Cu Kα radiation (λ = 1.54 Å) and a Lynxeye detector.

**NMR Spectroscopy.** Agilent MR 400 NMR spectrometer (9.4 T) was used to acquire ^119^Sn, ^1^H, ^13^C, and ^31^P measurements.

**STEM.** The samples were prepared on an ultrathin grid with 400 mesh, Cu (Ted Pella, Inc. 01822-F) which was wrapped with graphene on one side. The Sample was then drop-casted on the graphene side of the grid which is then sandwiched between two graphene layers using the other grid. The TEM grid was dried overnight in the antechamber of the glove box. The measurements were performed on a Thermo Fisher Themis Z STEM operating at 300 kV.

**SEM.** SEM was performed via a FEI Helios G4 CX electron microscope in scanning transmission mode operated at 18 kV using an ultrathin grid with 400 mesh, Cu (Ted Pella, Inc. 01822-F).

**Computational calculations**

All computational calculations were performed using the ORCA 6 code (version 6.0.0).^2^ The SnI_2_(R-NH_2_/R_3_’-P)_m/n_ adducts were obtained by an iterative docking procedure using the DOCKER algorithm at the GFN2-xTB/ALPB(Toluene)^3, 4^ level of theory, and the resulting structures were reoptimized at the density functional theory (DFT) M062X-D3/def2-TZVP+CPCM(Toluene) level.^5-8^ For the DFT calculations, relativistic corrections to the energies were applied by means of the ZORA scalar scheme.^9^ The Sn-N, Sn-P and Sn-I bond strength were characterized by the Intrinsic Bond Strength Index (IBSI) as implemented in the IMGPlot program ^10^ using the electron density calculated at the M062X-D3/def2-TZVP+CPCM(Toluene) level. In all the calculations, the neutral states of OLA and TOP were considered. The predicted absorption spectra were convoluted at the B3LYP-D3/def2-TZVP+CPCM(Toluene)^11-13^ level of theory on top of the ground state M062X-D3/def2-TZVP+CPCM(Toluene) ^5-8^ structure with gaussian functions with a full-width-at-half-maximum (FWHM) of 0.3 eV. The chemical structures and natural transition orbitals (NTOs) were rendered with Chemcraft.^14^ The stability of the dimeric and trimeric species was assessed as the energy difference between the optimized multimers and *n* times (n=2 for dimers, n=3 for trimers) the energy of the monomers divided by the number of monomer units in the species.

**III.** **Supporting figures/tables**

***
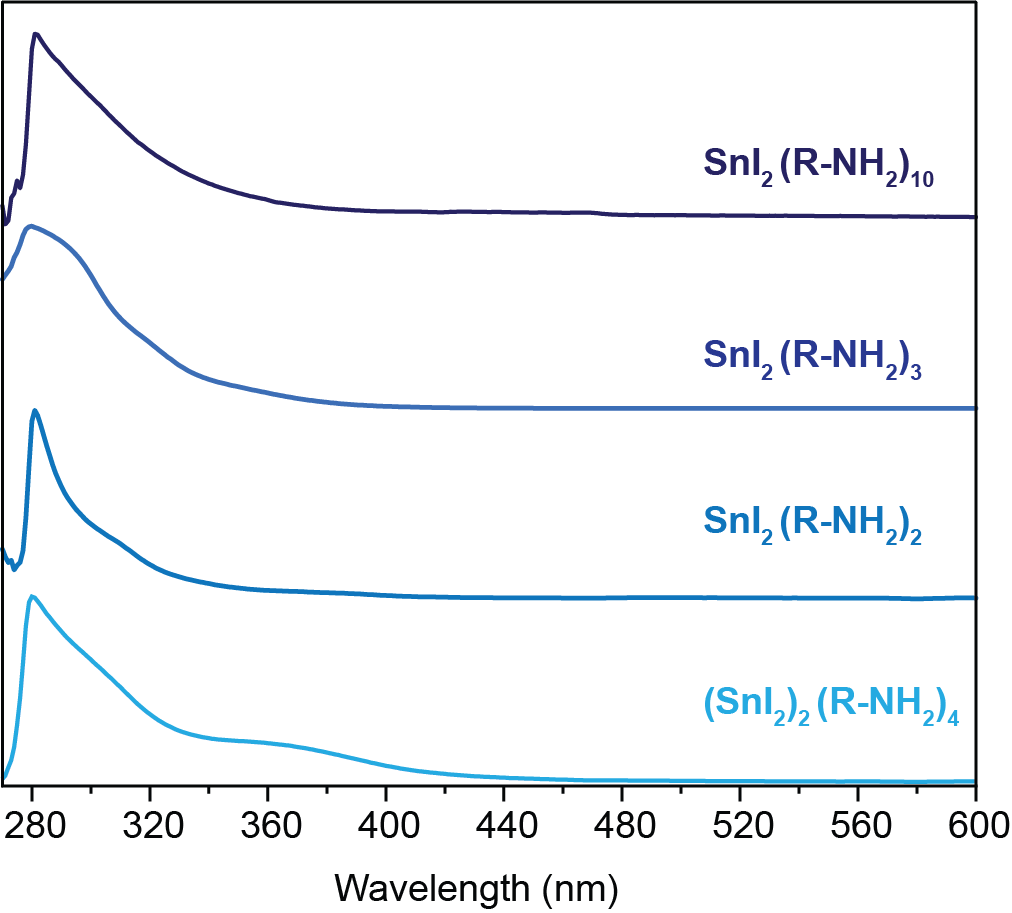
***

***Figure S1.*** *Absorbance of SnI_2_(R-NH_2_)_x_ complexes, expanded region to 270 nm.*


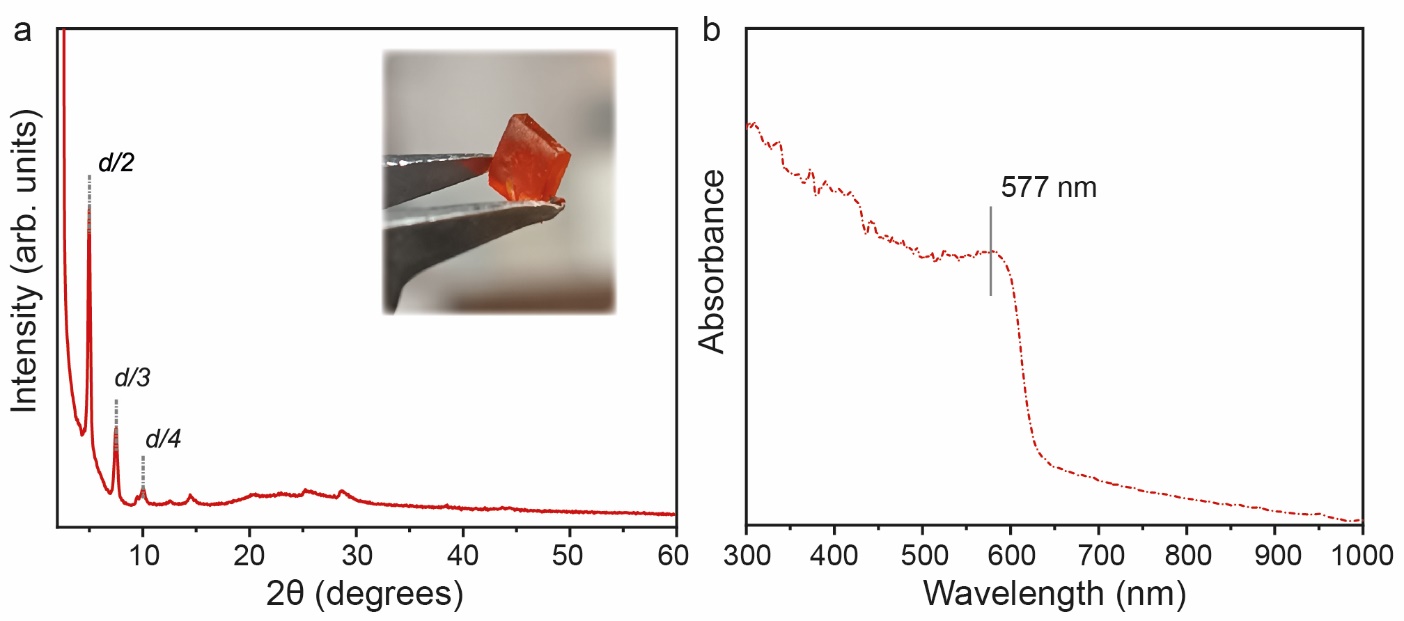


**Figure S2.** (a) XRD pattern of SnI_2_ + R-NH_2_ ,n=1 yellow solution drop-casted on a Si wafer forming a red crystallization product. Inset: A snapshot of red crystallization product on a glass substrate. (b) UV-Visible spectroscopy of a drop-casted red crystallization product in transmission mode with absorbance peak at 577 nm.


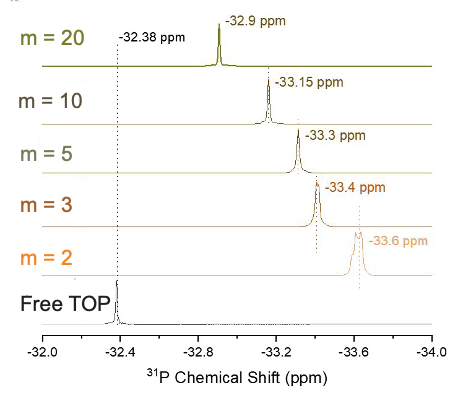


***Figure S3.*** *^31^P NMR spectra for different SnI_2_(R’_3_-P)_y_ solutions with a zoomed in chemical shift range from -34 ppm to -32 ppm.*


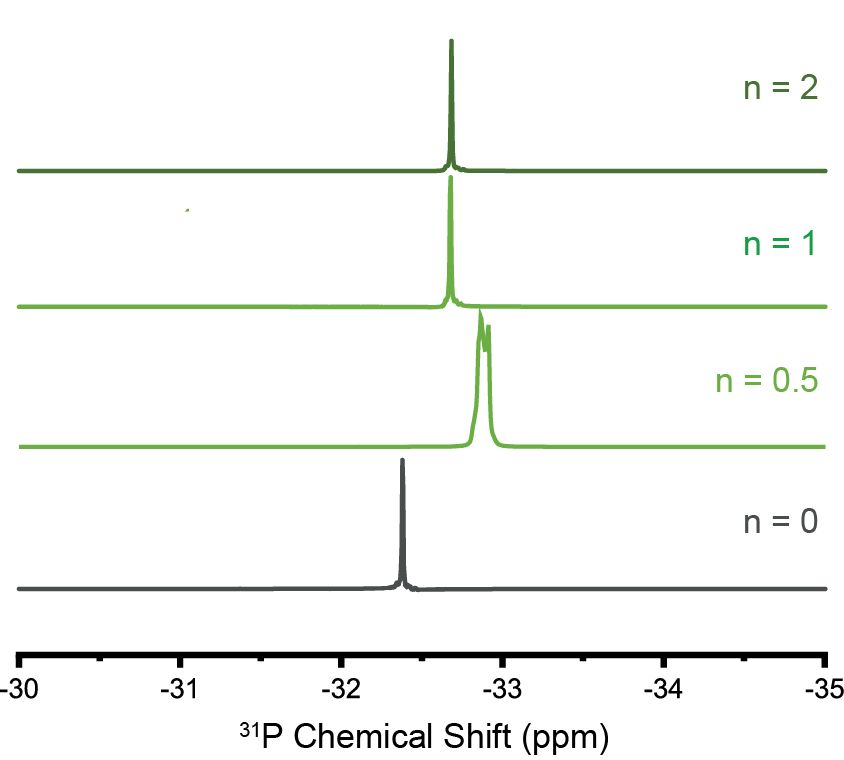


***Figure S4.*** *^31^P NMR spectra for different SnI_2_(R’_3_-P)_1_ (R-NH_2_)_x_ solutions with a zoomed in chemical shift range from -34 ppm to -32 ppm in (d).*

***
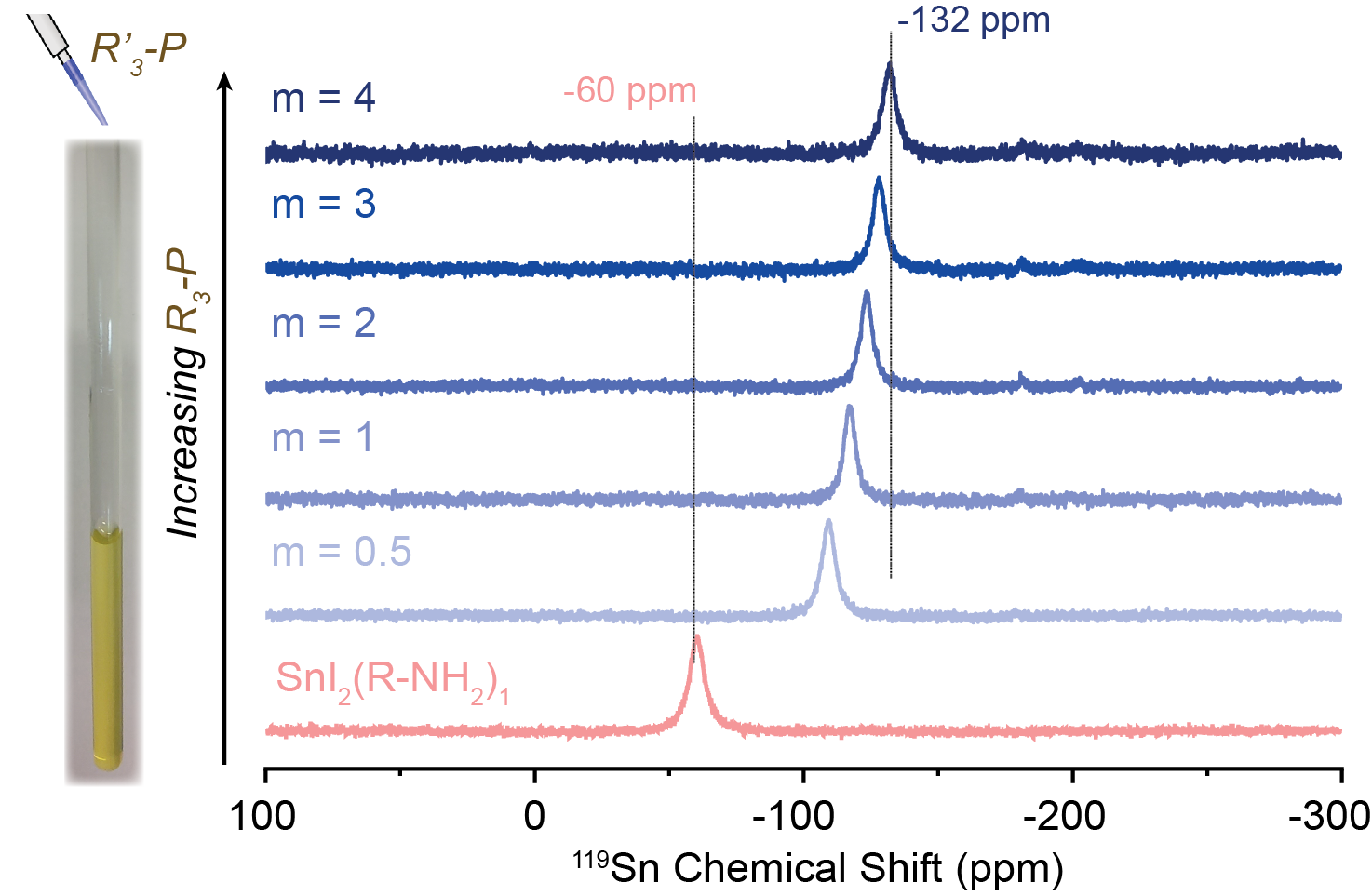
***

***Figure S5.*** *^119^Sn liquid-state NMR spectroscopy of SnI_2_(R-NH_2_)_1_ solutions with m.(R’_3_-P) in toluene-D8 respectively.*


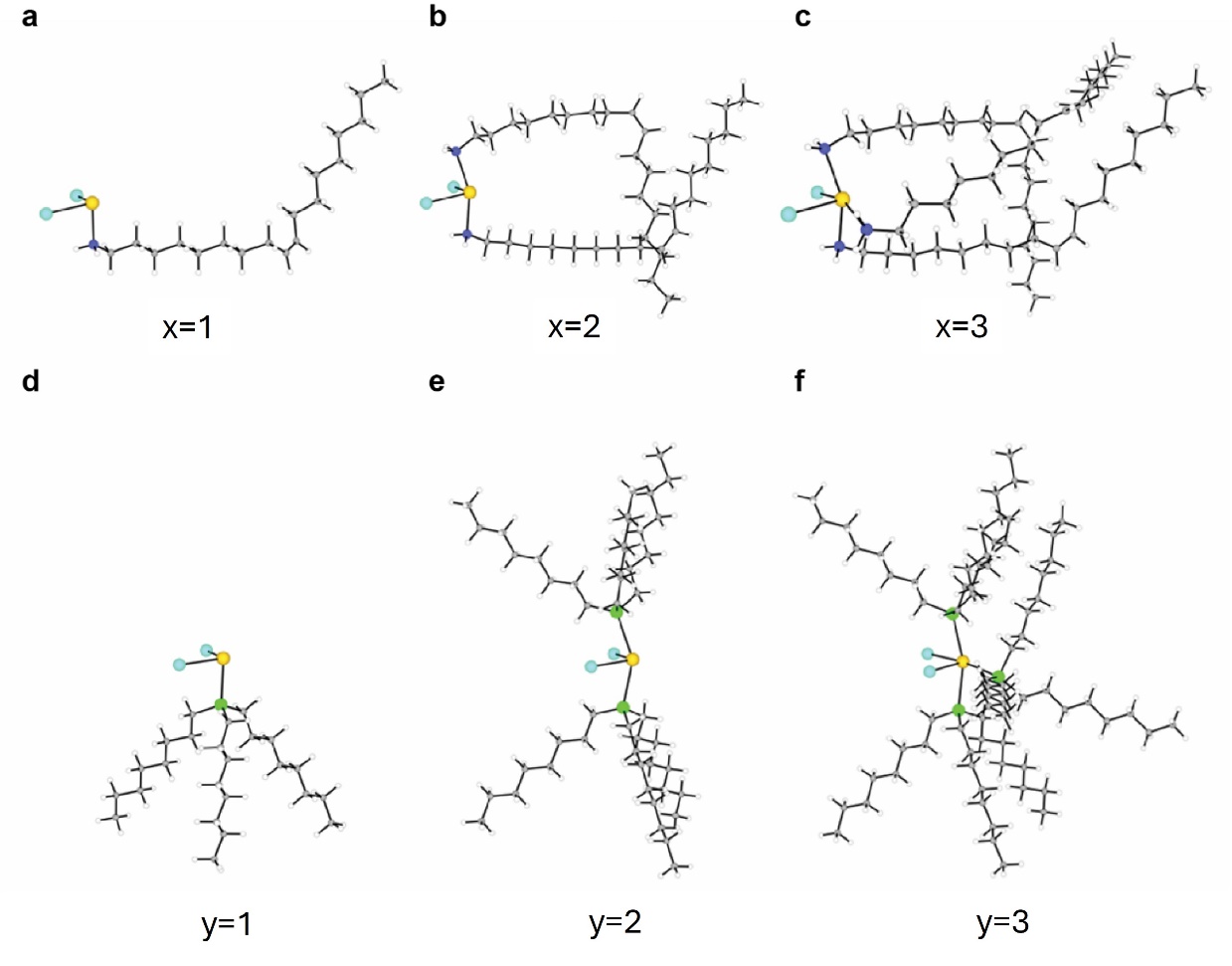


***Figure S6****. M062X/def2-TZVP+CPCM(Toluene) minimum-energy structures of the a-c: SnI_2_(R-NH_2_)_x_ complexes and d-f: SnI_2_(R_3_’-P)_y_ complexes obtained from the iterative GFN2-xTB docking procedure, with increasing values of n and m. Atom color code: Sn (yellow), N(blue), I (turquoise), C (grey), H (white).*

***Table S1****. M062X/def2-TZVP+CPCM(Toluene) SnI_2_(R-NH_2_)_x-1_-R-NH_2_ interaction energy (E_int_) and Sn-N IBSI values.*

| x | E_int_ (kcal/mol) | Sn-N IBSI |
| --- | --- | --- |
| 1 | -31.28 | 0.113 |
| 2 | -23.84 | 0.089 |
| 3 | -24.55 | 0.053 |

***Table S2****. M062X/def2-TZVP+CPCM(Toluene) SnI_2_(R_3_’-P)_x-1_-R_3_’-P interaction energy (E_int_) and Sn-P IBSI values.*

| x | E_int_ (kcal/mol) | Sn-P IBSI |
| --- | --- | --- |
| 1 | -36.96 | 0.097 |
| 2 | -17.73 | 0.050 |
| 3 | -18.89 | 0.048 |


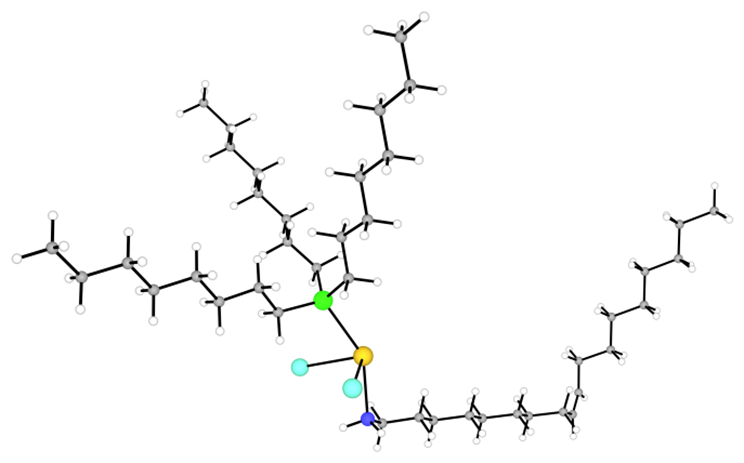


***Figure S7****. M062X/def2-TZVP+CPCM(Toluene) minimum-energy structure of the SnI_2_(R-NH_2_)(R_3_’-P) adduct. Atom color code: Sn (yellow), N (blue), I (turquoise), C (grey), H (white).*

***Table S3.*** *M062X/def2-TZVP+CPCM(Toluene) Sn-N and Sn-P IBSI values for the Sn(R-NH_2_/R_3_’-P)_x_^2+^ (x=1 and x=2, respectively) adducts and Sn-I IBSI values for the molecular SnI_2_.*

|  | **Sn-N/Sn-P** | | **Sn-I** |
| --- | --- | --- | --- |
|  | **x=1** | **x=2** |  |
| Sn(R-NH_2_)_x_^2+^ | 0.192 | 0.189 | - |
| Sn(R_3_^’^-P)_x_^2+^ | 0.156 | 0.153 | - |
| SnI_2_ | - | | 0.275 |


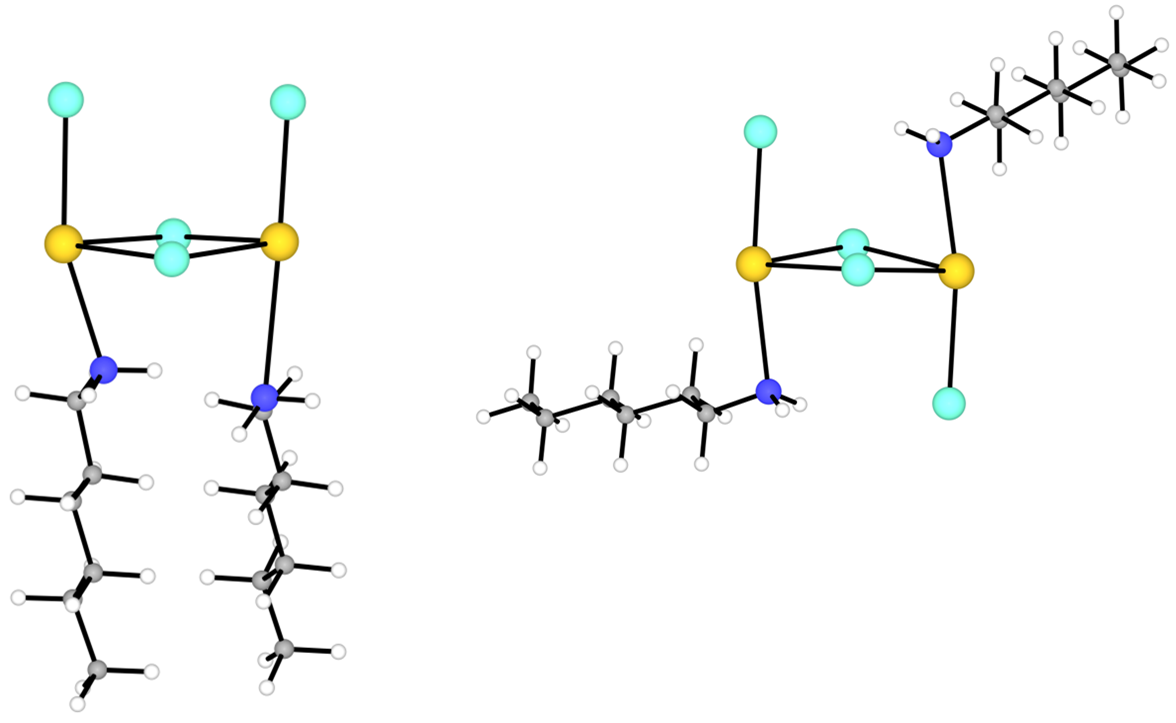


***Figure S8.*** *M062X/def2-TZVP+CPCM(Toluene) minimum-energy structure of the (SnI_2_)_2_(R-NH_2_)_2_ adducts: cis (left), trans (right). Atom color code: Sn (yellow), N (blue), I (turquoise), C (grey), H (white). The OLA chain was cut for simplicity.*


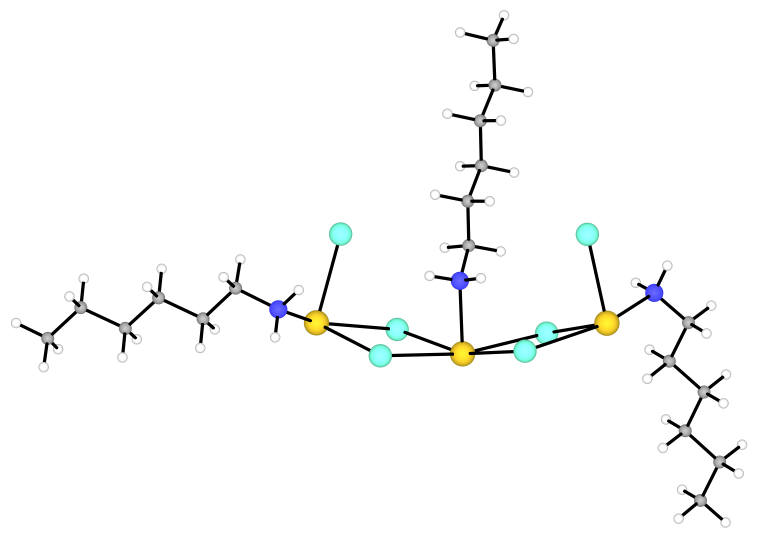


***Figure S9.*** *M062X/def2-TZVP+CPCM(Toluene) minimum-energy structure of the (SnI_2_)_3_(R-NH_2_)_3_ adduct. Atom color code: Sn (yellow), N (blue), I (turquoise), C (grey), H (white). The OLA chain was cut for simplicity.*

*
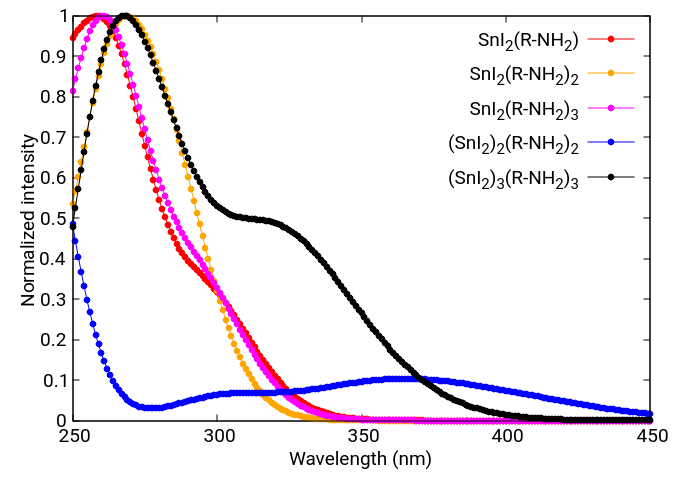
*

***Figure S10****. B3LYP-D3/def2-TZVP+CPCM (Toluene) predicted absorption spectra for the different R-NH_2_-based adducts.*


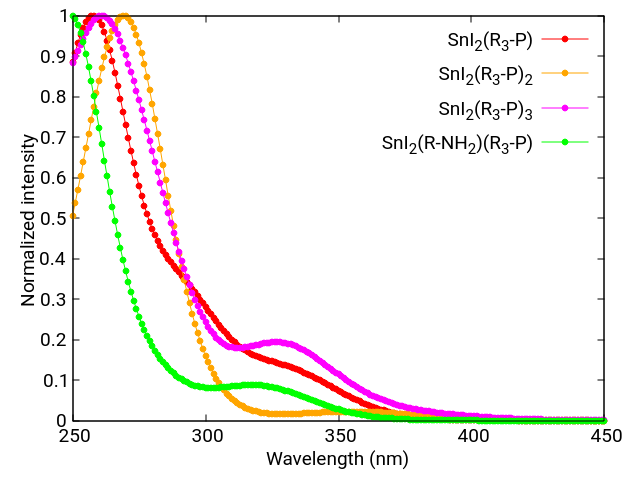


***Figure S11****. B3LYP-D3/def2-TZVP+CPCM (Toluene) predicted absorption spectra for the different R_3_’-P (TOP)-based adducts and for that the R-NH_2_/R_3_’-P 1:1 ratio SnI_2_(R-NH_2_)(R_3_’-P).*


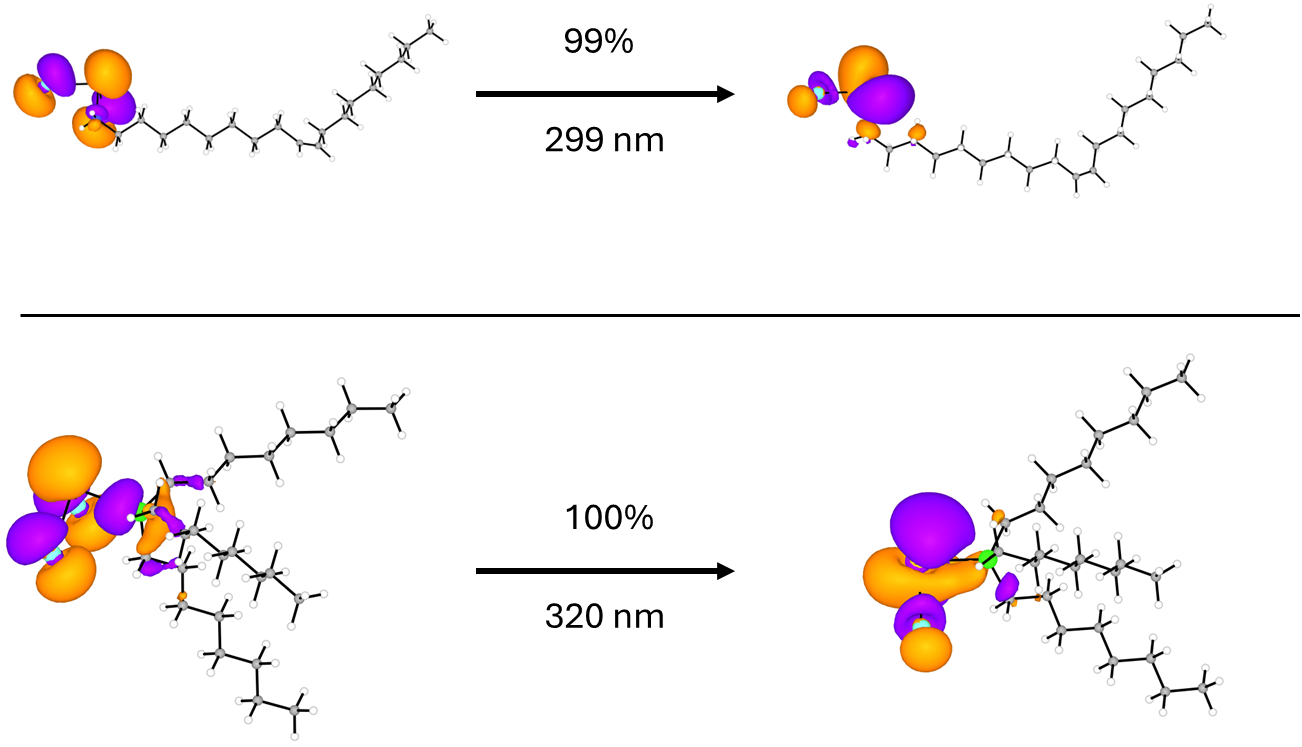


***Figure S12****. B3LYP-D3/def2-TZVP+CPCM (Toluene) natural transition orbitals (NTOs) involved in the most intense electronic transition around 300 nm for both SnI_2_(R-NH_2_) (top) and SnI_2_(R_3_’_-_P) (bottom). Atom color code: Sn (yellow), N (blue), I (turquoise), C (grey), H (white). Rendering isovalue: 0.03.*


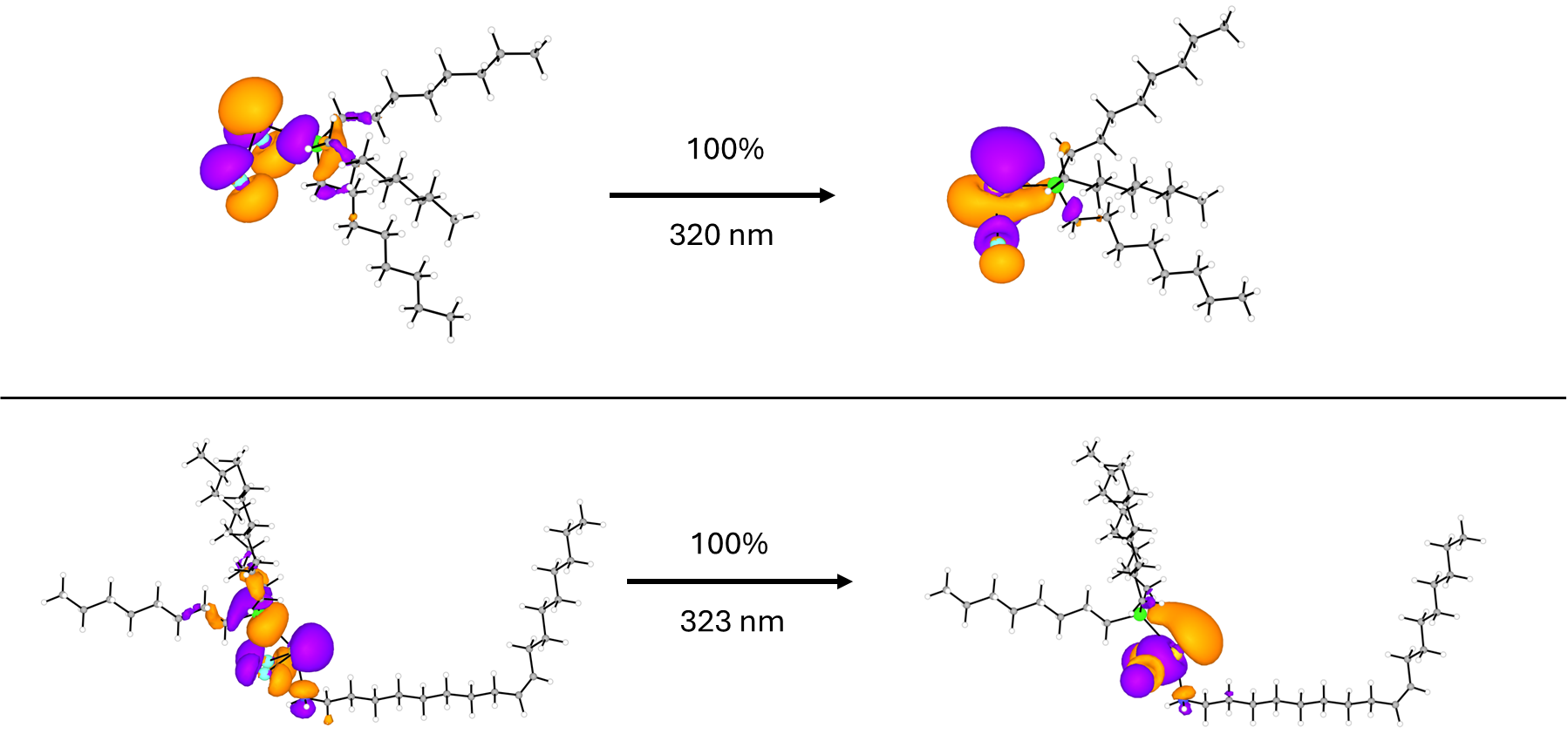


***Figure S13****. B3LYP-D3/def2-TZVP+CPCM (Toluene) natural transition orbitals (NTOs) involved in the most intense electronic transition around 330 nm both SnI_2_(R_3_’-P) (top) and SnI_2_(R-NH_2_)(R_3_’-P). Atom color code: Sn (yellow), N (blue), I (turquoise), C (grey), H (white). Rendering isovalue: 0.03.*


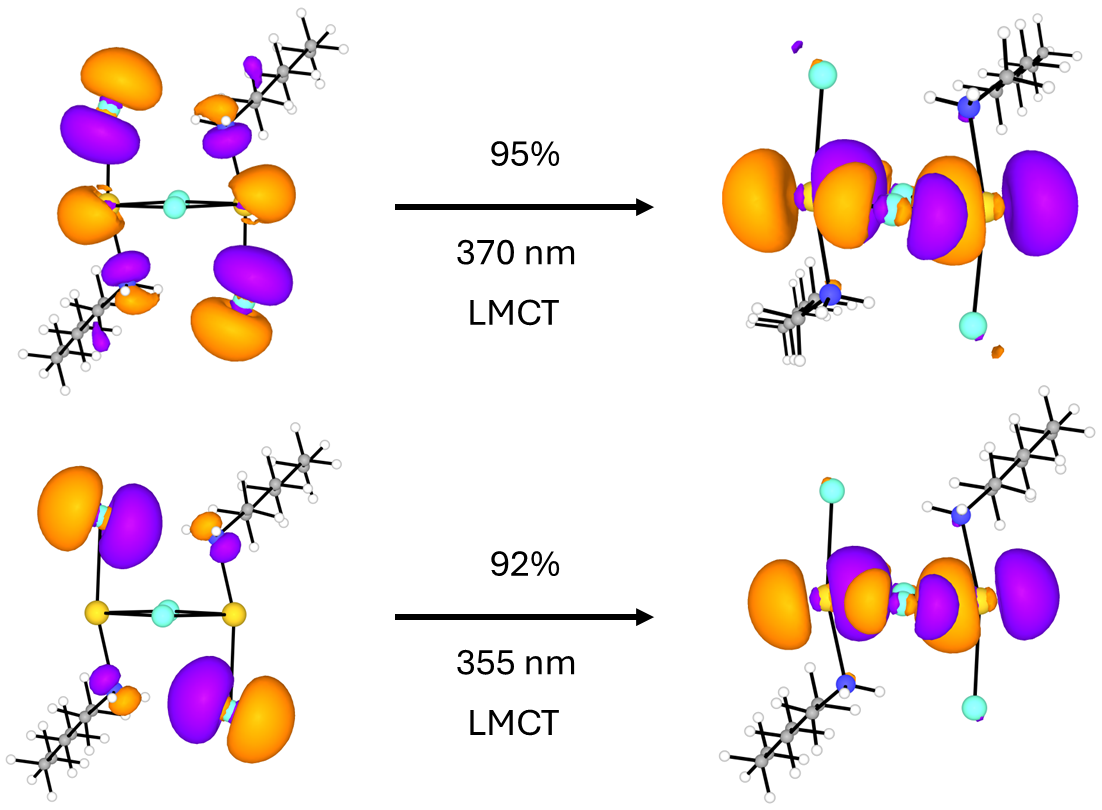


***Figure S14****. B3LYP-D3/def2-TZVP+CPCM (Toluene) natural transition orbitals (NTOs) involved in the most intense electronic transitions in the 300-400 nm range for the (SnI_2_)_2_(R-NH_2_)_2_ trans dimer. Atom color code: Sn (yellow), N (blue), I (turquoise), C (grey), H (white). Rendering isovalue: 0.03.*

*
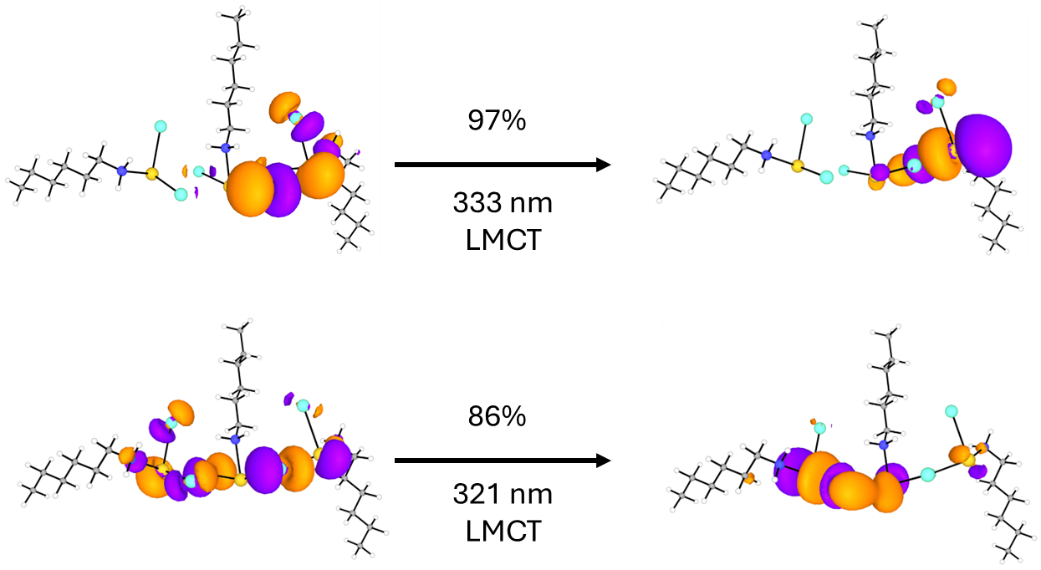
*

***Figure S15****. B3LYP-D3/def2-TZVP+CPCM (Toluene) natural transition orbitals (NTOs) involved in the most intense electronic transitions in the 300-400 nm range for the (SnI_2_)_3_(R-NH_2_)_3_ trimer. Atom color code: Sn (yellow), N (blue), I (turquoise), C (grey), H (white). Rendering isovalue: 0.03.*


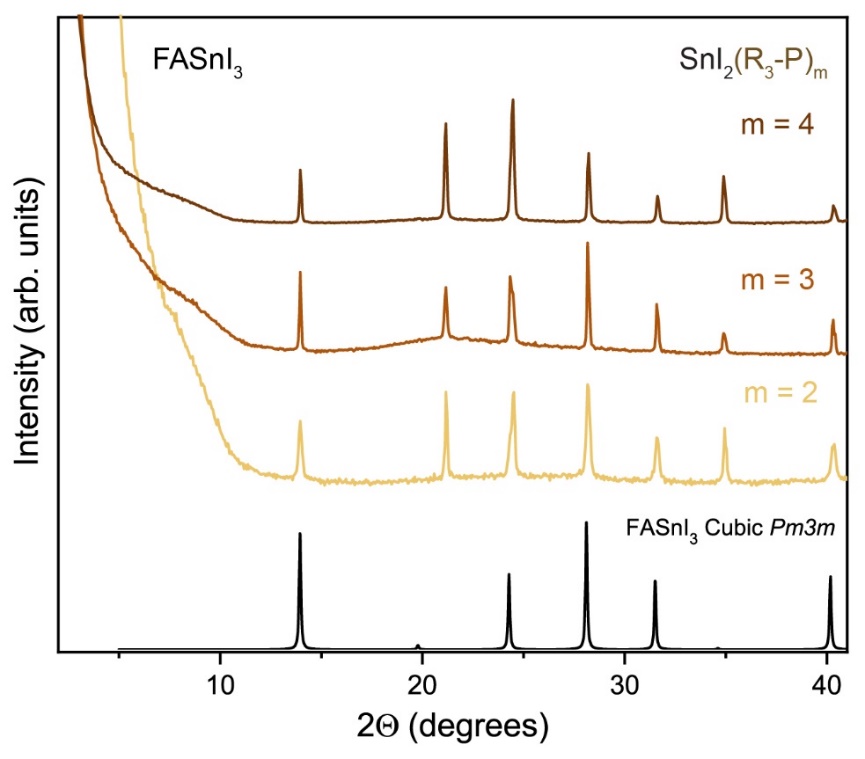


***Figure S16.*** *XRD pattern of the FASnI_3_ formed in the different SnI_2_(R_3_^’^-P)_y_ solutions plotted with the bulk reference FASnI_3_ cubic Pm3m.^15^*


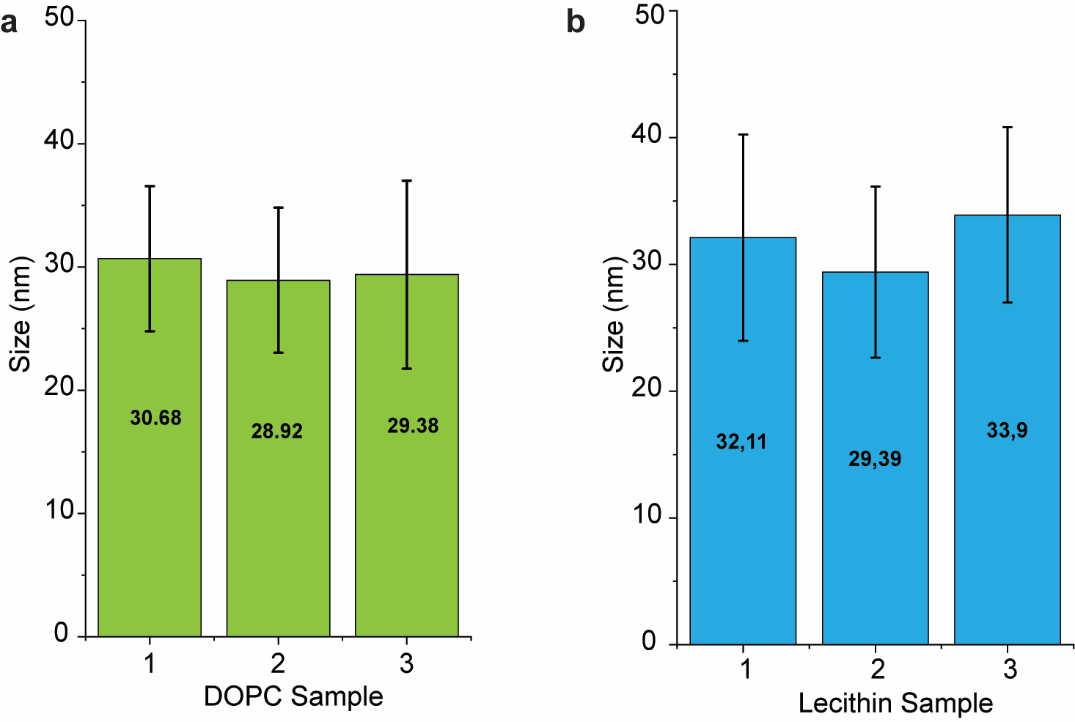


***Figure S17****. Size distribution for a) DOPC and b) Lecithin capped FASnI_3_ NCs.*

***References:***

(1) Theobald, D. W.; Smith, J. C. Acid iodides. *Chem. & Ind* **1958**, 1007-1009.

(2) Neese, F. Software update: The ORCA program system—version 6.0. *Wiley Interdiscip. Rev.: Comput. Mol. Sci.* **2025**, *15* (2), e70019.

(3) Bannwarth, C.; Ehlert, S.; Grimme, S. GFN2-xTB—An accurate and broadly parametrized self-consistent tight-binding quantum chemical method with multipole electrostatics and density-dependent dispersion contributions. *J. Chem. Theory Comput.* **2019**, *15* (3), 1652-1671.

(4) Ehlert, S.; Stahn, M.; Spicher, S.; Grimme, S. Robust and efficient implicit solvation model for fast semiempirical methods. *J. Chem. Theory Comput.* **2021**, *17* (7), 4250-4261.

(5) Zhao, Y.; Truhlar, D. G. The M06 suite of density functionals for main group thermochemistry, thermochemical kinetics, noncovalent interactions, excited states, and transition elements: two new functionals and systematic testing of four M06-class functionals and 12 other functionals. *Theor. Chem. Acc.* **2008**, *120* (1), 215-241.

(6) Grimme, S.; Antony, J.; Ehrlich, S.; Krieg, H. A consistent and accurate ab initio parametrization of density functional dispersion correction (DFT-D) for the 94 elements H-Pu. *J. Chem. Phys.* **2010**, *132* (15).

(7) Schäfer, A.; Huber, C.; Ahlrichs, R. Fully optimized contracted Gaussian basis sets of triple zeta valence quality for atoms Li to Kr. *J. Chem. Phys.* **1994**, *100* (8), 5829-5835.

(8) Garcia‐Ratés, M.; Neese, F. Effect of the Solute Cavity on the Solvation Energy and its Derivatives within the Framework of the Gaussian Charge Scheme. *J Comput Chem.* **2020**, *41* (9), 922-939.

(9) Van Lenthe, E. v.; Snijders, J.; Baerends, E. The zero order regular approximation for relativistic effects: the effect of spin-orbit coupling in closed shell molecules. *J. Chem. Phys.* **1996**, *105*, 6505-6516.

(10) Lefebvre, C.; Klein, J.; Khartabil, H.; Boisson, J. C.; Hénon, E. IGMPlot: A program to identify, characterize, and quantify molecular interactions. *J. Comput. Chem.* **2023**, *44* (20), 1750-1766.

(11) Lee, C.; Yang, W.; Parr, R. G. Development of the Colle-Salvetti correlation-energy formula into a functional of the electron density. *Physical review B* **1988**, *37* (2), 785.

(12) Vosko, S. H.; Wilk, L.; Nusair, M. Accurate spin-dependent electron liquid correlation energies for local spin density calculations: a critical analysis. *Can. J. Phys.* **1980**, *58* (8), 1200-1211.

(13) Stephens, P. J.; Devlin, F. J.; Chabalowski, C. F.; Frisch, M. J. Ab initio calculation of vibrational absorption and circular dichroism spectra using density functional force fields. *J. Phys. Chem.* **1994**, *98* (45), 11623-11627.

(14) *Chemcraft - graphical software for visualization of quantum chemistry computations. Version 1.8, build 682.* [*https://www.chemcraftprog.com*](https://www.chemcraftprog.com)

(15) Morana, M.; Wiktor, J.; Coduri, M.; Chiara, R.; Giacobbe, C.; Bright, E. L.; Ambrosio, F.; De Angelis, F.; Malavasi, L. Cubic or Not Cubic? Combined Experimental and Computational Investigation of the Short-Range Order of Tin Halide Perovskites. *J. Phys. Chem. Lett.* **2023**, *14* (8), 2178-2186.
